# Comparison of Automatic Segmentation and Preprocessing Approaches for Dynamic Total-Body 3D Pet Images with Different Pet Tracers

**DOI:** 10.1007/s10278-025-01540-4

**Published:** 2025-05-27

**Authors:** Maria K. Jaakkola, Marcela Xiomara Rivera Pineda, Rafael Díaz, Maria Rantala, Anna Jalo, Henri Kärpijoki, Teemu Saari, Teemu Maaniitty, Thomas Keller, Heli Louhi, Saara Wahlroos, Merja Haaparanta-Solin, Olof Solin, Jaakko Hentilä, Jatta S. Helin, Tuuli A. Nissinen, Olli Eskola, Johan Rajander, Juhani Knuuti, Kirsi A. Virtanen, Jarna C. Hannukainen, Francisco López-Picón, Riku Klén

**Affiliations:** 1 Turku PET Centre, University of Turku, Åbo Akademi University, and Turku University Hospital, Turku, Finland; 2 Biomedical Imaging, Åbo Akademi University, Turku, Finland; 3 MediCity Research Laboratory, University of Turku, Turku, Finland; 4 PET Preclinical Laboratory, Turku PET Centre, University of Turku, Turku, Finland; 5 Department of Clinical Physiology, Nuclear Medicine and PET, Turku University Hospital, Turku, Finland; 6 Radiopharmaceutical Chemistry Laboratory, Turku PET Centre, University of Turku, Turku, Finland; 7 Department of Chemistry, University of Turku, Turku, Finland; 8 Accelerator Laboratory, Turku PET Centre, Åbo Akademi University, Turku, Finland; 9 Department of Chemistry, University of Turku, Turku, Finland; 10 Faculty of Sport and Health Sciences, University of Jyväskylä, Jyväskylän, Finland

**Keywords:** Segmentation, Comparison, PET

## Abstract

Segmentation is a routine step in PET image analysis, and few automatic tools have been developed for it. However, excluding supervised methods with their own limitations, they are typically designed for older, small images and the implementations are no longer publicly available. Here, we test if different commonly used building blocks of the automatic methods work with large modern total-body PET images. Dynamic total-body images from five different datasets are used for evaluation purposes, and the tested algorithms cover wide range of different preprocessing approaches and unsupervised segmentation methods. The validation is done by comparing the obtained segments to manually drawn ones using Jaccard index, Dice score, precision, and recall as measures of match. Out of the 17 considered segmentation methods, only 6 were computationally usable and provided enough segments for the needs of this study. Among these six feasible methods, hierarchical clustering and HDBSCAN had systematically the lowest Jaccard indices with the manual segmentations, whereas both GMM and *k*-means had median Jaccards of 0.58 over different organ segments and data sets. GMM outperformed *k*-means in human data, but with rat images, the two methods had equally good performance *k*-means having slightly stronger precision and GMM recall. We conclude that most of the commonly used unsupervised segmentation methods are computationally infeasible with the modern PET images, classical clustering algorithms *k*-means and especially Gaussian mixture model being the most promising candidates for further method development. Even though preprocessing, particularly denoising, improved the results, small organs remained difficult to segment.

**Supplementary Information:**

The online version contains supplementary material available at 10.1007/s10278-025-01540-4.

## Introduction

Segmentation is a routine step when analysing positron emission tomography (PET) images, and it can be done either manually, or with supervised or unsupervised automatic methods. Especially in clinical use, manual segmentation is still the most common approach. However, it is time-consuming, subjective, and it makes utilising the time aspect of nowadays mainstream dynamic images very difficult.

Supervised machine learning (ML) approaches are an appealing option for segmenting large dynamic human total-body images. However, PET is not the best-suited imaging modality for ML, because different radiotracers behave very differently from each other, which makes the images immensely heterogeneous. Another issue is the availability of training data. Despite the challenges, several specialised ML-based methods have been proposed [1–3]. However, none of them segments the whole image, but focus on certain areas (tumours, organs, etc.) according to the utilised training data. Often, PET is combined with computer tomography (CT) images in different ML approaches [4–7], though also these tools cannot do a complete segmentation, but focus on certain parts, tumours being the most common application. The strength of unsupervised methods as compared to training data based ML methods is their capability to segment the whole area of any PET images regardless of the scanned area, organism, or used tracer. While the available deep learning methods are not for general purpose, their strengths include good performance on their specific goal and computationally light use from the end-user point of view. The training phase requires plenty of resources from running time and hardware perspective, but after that, the actual use of such methods is typically fast and doable even with a mediocre computer. Yousefiri et al. provide a great summary of strengths and weaknesses of ML-based segmentation methods in the context of PET images [8]. In this study, we focus on unsupervised methods.

Several studies introducing unsupervised segmentation approaches designed for dynamic PET images have been published. Majority of the examples we found are based on clustering combined with suitable preprocessing of the data. For example, Zbib et al. [9] introduce a spectral clustering based approach with data projection and automatic parameter detection, and Kimura et al. [10] normalise time activity curves (TACs) prior to principal component analysis and clustering in order to reduce noise and simplify the data. *K*-means is a popular clustering method for segmenting dynamic PET images, likely because it is computationally light and fast compared to many other clustering methods [11]. Kim et al. [12] combine *k*-means clustering and region growing in order to utilise both spatial and temporal information in the segmentation, and Wong et al. [13] cluster total-body images rather than brain regions with tweaked *k*-means. Guo et al. [14] take into account the challenges presented by the huge number of voxels and use coarse pre-clustering prior to the more intensive clustering to segment brain regions. Besides clustering-based approaches, also, contouring has been used for segmenting dynamic PET images in the literature. Maroy et al. [15] propose a sophisticated approach which first identifies the centre of each volume of interest (VOI) as a neighbourhood with low noise variance, and then uses them to segment the whole image with the minimal energy path active contouring suggested by Cohen and Kimmel [16]. Their method is designed for total-body images. Another contouring-based method was proposed by Shepherd and Owenius [17]. Their approach is designed for cancer studies and treats TACs as stochastic processes. An interesting exception is the study by Cheng-Liao et al. [18] as their approach is based on level set method. Notably, these methods are rather old and the implementations are no longer available. Also, most of these methods are designed for small-size images and for computational reasons are not usable with modern dynamic human total-body images including tens of millions of voxels [11]. The novel method development has mainly focused on supervised training data based methods, and it is unknown if the previously published unsupervised methods would be usable on large modern images, if the implementations were available. The reported performances are not easy to compare due to different evaluation metrics and varying level of difficulty, but for example Kim et al. report mean Dice scores of 0.40–0.67 for segmenting white and grey matter from brain images, whereas Cheng-Liao et al. report Dice scores of 0.03–0.89 for different organs of mice. In addition, Weisman et al. have compared different traditional methods’, such as clustering approaches, thresholding, and region growing, as well as machine learning approaches’ (e.g. U-Net and DeepMedic), capability to detect lymphoma lesions from PET/CT images [19]. The obtained median Dice scores were typically around 0.6 for the evaluated methods. Similar third-party comparison was earlier carried out by Dewalle-Vignion et al. using smaller set of methods providing mean Jaccard index of 0.45 at best [20]. Notably, the reported accuracies of these two comparison studies are very similar as Jaccard index of 0.45 converted into Dice score is 0.62.

**Table 1:** Summary of the utilised datasets

	F-DPA	UCB-J	FDG	FDG human	H2O human
Images	36	42	41	24	5
Mean weight	465 g	479 g	487 g	71.5 kg UPDATE	84.2 kg
Tracer	[$^{18}$F]F-DPA	[$^{11}$C]UCB-J	[$^{18}$F]FDG	[$^{18}$F]FDG	[$^{15}$O]H$_{2}$O
Mean dose	20.80 MBq	30.36 MBq	20.77 MBq	104.73 MBq UPDATE	335.6 MBq
Time frames	51	51	50	13	24

Here, we provide an empirical comparison of basic building blocks of the previously used segmentation methods using modern dynamic total-body PET images. To ensure that our conclusion generalise as well as possible, we test the methods on three otherwise similar rat datasets, but scanned with different tracers, namely [$^{18}$F]F-DPA, [$^{11}$C]UCB-J, and [$^{18}$F]FDG. Our codes are available at https://github.com/rklen/Preprocessing_and_Segmentation_Evaluation_PET. We first filter out the basic unsupervised segmentation methods that cannot be used with large total-body images for computational reasons. Then, we test the remaining methods with several preprocessing approaches using a subset of images from each dataset. Among the standard preprocessing steps, we test different denoising, scaling, and dimensionality reduction methods. Then, we define a suitable number of segments and use the best pipeline to analyse the remaining images. We also run the best pipelines first for few dynamic human total-body images scanned using two different tracers, and then the feasible approaches for larger human dataset with manual segments available for comparison. In addition, we briefly test several related aspects suggested in the literature or otherwise relevant, such as excluding early time points, splitting the image into smaller subimages, and usage of raw intensity data versus standardised uptake values (SUV).

## Materials and Methods

### Data

**Fig. 1:**
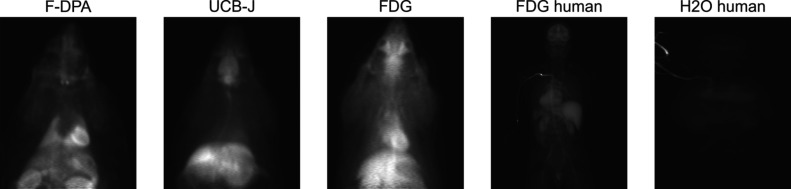
Example images from each dataset illustrate sum of activity levels over time and height dimension due to 2D visualisation

We used three datasets including dynamic total-body 3D PET images of rats and two datasets containing dynamic total-body 3D PET images of humans. All rat datasets were produced at Turku PET centre using PET/CT scanner Inveon Multimodality Platform by Siemens Molecular Imaging. The human subjects were scanned with Siemens’ PET/CT scanner Vision Quadra Edge and all participants gave their written consent. Table 1 summarises the utilised datasets, and Fig. 1 visualises example images from them. The CT images are not utilised in this study. The main difference between the rat datasets is that different tracer is used in each of them. Due to the usage of the same scanner, all rat images consist of 128$\cdot$128$\cdot$159 = 2,605,056 voxels. The spatial dimensions of the human images were 440$\cdot$440$\cdot$354 = 68,534,400 voxels, but the [$^{15}$O]H$_{2}$O human images were reduced to 220$\cdot$220$\cdot$380 = 18,392,000 voxels prior to this study. However, in case of clustering algorithms, not all the voxels were used for segmentation. The voxels with mean intensity over time below the average were defined as background and were excluded prior to clustering analyses. Methods designed specifically for image segmentation required the whole image as input. Notably, for most of the tests, only clustering methods were usable.

The first dataset includes 36 images of healthy Fischer344 rats scanned using radiotracer [$^{18}$F]F-DPA [21]. The mean injected dose was 20.80 MBq (standard deviation 0.78 MBq) and the rats weighted 465 g on average (standard deviation 125 g). The scans were designed to have 51 time frames ($30\cdot 10$ s, $15\cdot 60$ s, $4\cdot 300$ s, $2\cdot 600$ s). The animal study was approved by the State Provincial Office of Southern Finland (licence ESAVI-33741-2019). This dataset is further referred to as F-DPA data.

The second dataset comprises 42 PET images of rats labelled with radiotracer [$^{11}$C]UCB-J [22]. The mean injected dose was 30.36 MBq (standard deviation 3.00 MBq). The scanned rats belonged to the Fischer344 strain and had an average weight of 479 g (standard deviation 120 g). Also, these scans had 51 time frames ($30\cdot 10$ s, $15\cdot 60$ s, $4\cdot 300$ s, $2\cdot 600$ s). This study was done under the same licence (ESAVI-33741-2019) than the F-DPA data. The abbreviation used for this dataset is UCB-J data.

The third dataset was labelled with radiotracer 2-Deoxy-2-[$^{18}$F]fluoroglucose ([$^{18}$F]FDG), and it contains 43 images of healthy male Sprague-Dawley rats. The mean tracer dose was 20.77 MBq (standard deviation of 1.16 MBq) and the scanned rats weighted 487 g on average (standard deviation 87 g). This study included 50 time frames ($30\cdot 10$ s, $15\cdot 60$ s, $5\cdot 300$ s). The animal study was approved by the State Provincial Office of Southern Finland with the licence number of ESAVI-4080-2019. For the rest of this study, this dataset is called FDG data.

Our first human dataset included 24 dynamic total-body PET images of humans, and it is called FDG human data in this study. All human subjects were healthy according to routine laboratory tests, oral glucose tolerance test, and medical examination. The weights of the individuals were 69.7 kg on average, and the mean injected tracer dose was 107.25 MBq. The scan was started immediately after bolus injection of [$^{18}$F]FDG. The scans had 13 time frames ($1\cdot 60$ s, $6\cdot 30$ s, $1\cdot 60$ s, $3\cdot 300$ s, $2\cdot 600$ s). The reference number of the ethical committee decision related to the FDG human data was 14/1801/2022 (Hospital District of South-Western Finland).

The second human dataset was scanned using radiotracer [$^{15}$O]H$_{2}$O, and here, it is referred to as H2O human data. We used five images of patients who underwent PET myocardial perfusion imaging during adenosine vasodilator stress for evaluation of suspected or known coronary artery disease. Two of the subjects were females and three were males. Their weights were 90 kg, 94 kg, 79 kg, 85 kg, and 73 kg, and the corresponding tracer doses were 339 MBq, 350 MBq, 353 MBq, 315 MBq, and 321 MBq. The scans consisted of 24 time frames ($14\cdot 5$ s, $3\cdot 10$ s, $3\cdot 20$ s, $4\cdot 30$ s), and the reference number of the relevant ethical committee decision was 22/1801/2022.

The manual segmentation of the rat images serving as gold standard for validating the automatic segmentation was drawn using version 2.10 of Carimas software [23]. The first step to draw the manual segments was to obtaining training from a biologist with over 15 years of experience on rat models and PET image analysis. Then, each rat dataset was segmented by a different person following the instructions from the expert. Finally, the expert inspected the ready segments and possibly suggested corrections, which were implemented. From F-DPA data PET images, five volumes of interest (VOIs) were segmented: brain, heart, lungs, pituitary gland, and thyroid glands. From the UCB-J data PET images, brain, kidneys, and liver were segmented, and from FDG data, the segmented VOIs were brain, heart, and kidneys. These VOIs are heterogeneous considering their size and tracer uptake. The liver segmented from UCB-J data was the largest one (34,191 voxels on average), and pituitary gland and thyroid glands from F-DPA data were the smallest ones (23 and 57 voxels on average). The tracer uptake was particularly high in the liver and the brain in UCB-J data and in the heart and the thyroid glands in F-DPA data. On the other hand, the brain was very difficult to detect with visual inspection from the F-DPA and FDG PET images due to low tracer uptake. Refer to Section 1 of Supplementary text for visualisation and further details about the manually segmented VOIs. Among the FDG human dataset, 22 out of 24 images were manually segmented according to instructions of an expert with a degree on human anatomy. The segmented organs were the heart, aorta, kidneys, liver, lungs, and brain. For the H2O human dataset, we do not have manual segmentations, but they serve as test data to evaluate computational feasibility of the identified best methods on very large images together with three images from the FDG human data.

With all rat datasets, we briefly tested if using standardised uptake values (SUVs) instead of raw intensities affects the clustering results. As it did not have high impact on the accuracy of the results (Section 2 of Supplementary text), we continued our analyses with raw data to minimise the data processing.

### Tested Segmentation Methods

Our selection criteria for methods evaluated in this comparison were as follows: (1) there has to be an implementation of the method freely available, (2) the method should provide different labels for different segments in the image without considerable manual tweaking or post processing, (3) the method has to be able to analyse 4D data of grey-scale images, and (4) the method should operate in an unsupervised manner and not be limited to specific organs, biological conditions, or species. We considered 17 methods and 6 out of them were usable with full size data (Table 2). We claimed a method as usable, if we managed to run it without errors and it took less than 2 h to segment one image using a computer with 16GB of RAM and Intel Pentium Gold processor G6405T (CPU3.50GHz). Preprocessing of the image was not counted towards the two hours cutoff. Notably, this cutoff was selected based on our patience. Running time and hardware requirements from clinical point of view are addressed in section Discussion and conclusions. Clustering algorithm ‘Density-Based Spatial Clustering of Applications with Noise’ (DBSCAN) and segmentation method ‘Simple Linear Iterative Clustering’ (slic) were fast enough to run with the default parameters, but those did not yield meaningful results (all voxels were labelled as noise or all segments were of regular rectangular shape). Different parameter choices caused our computer to crash (DBSCAN) or the running time to expand above our cutoff (slic); thus, we claimed also those methods as computationally infeasible. The usable methods only included clustering methods.

**Table 2:** Segmentation methods considered for this comparison

Method	Package	Included/excluded
Affinity propagation	sklearn	Memory error
BIRCH	sklearn	Memory error
DBSCAN	sklearn	Computer crashed
Fuzzy *c*-means	fcmeans	Included
GMM	sklearn	Included
HDBSCAN	hdbscan	Included
Hierarchical	fastcluster	Included
*k*-means	sklearn	Included
Mean shift	sklearn	Time cutoff
Mini-batch *k*-means	sklearn	Included
morphACWE	skimage	Only 2 segments
morphGAC	skimage	Only 2 segments
OPTICS	sklearn	Time cutoff
Random walker	skimage	Time cutoff
Slic	skimage	Time cutoff
Spectral	sklearn	Memory error
Watershed	skimage	Time cutoff

The first column indicates the method name (abbreviation used in case of long names), the second column tells the utilised Python package implementing the method, and the last column states if the method was used or why it was excluded from this study

### Test Design

In this study, we first test different preprocessing approaches using 10 randomly selected images from each rat dataset with manual segmentations available. In the preprocessing tests, all the six segmentation methods listed above, namely hierarchical clustering, Gaussian mixture model (GMM), *k*-means, mini-batch *k*-means, fuzzy *c*-means, and hierarchical DBSCAN (HDBSCAN), were used with 30 clusters. Notably, HDBSCAN is fully automatic and does not take even the number of clusters from the end user. Then, we evaluate different cluster numbers from interval 15–45. After this, we segment the remaining images, including the human data, using the best-performing preprocessing approaches and cluster number, and compare the performances of different segmentation methods in different VOIs. For the human images, the number of clusters was set to 150 due to the size difference of the rat and human images. Finally, we briefly test if the computationally infeasible methods would be promising if they were usable in practice.

Through this study, we use Jaccard index to measure how well each VOI was segmented. Jaccard index is a measure of similarity of two sets, and it is defined as the ratio of their intersection and their union:

1 $$\begin{aligned} Jaccard(A,B) = \frac{|A \cap B|}{|A \cup B|}, \end{aligned}$$

where *A* is the set of voxels belonging to the cluster(s) representing the analysed VOI, and *B* is the set of voxels in the manually segmented VOI. Notations $|A \cap B|$ and $|A \cup B|$ indicate the number of voxels in the intersection and union of *A* and *B*, respectively. For each VOI, the cluster or their combination with the highest Jaccard index was selected to represent it. A perfect segmentation would yield Jaccard index 1, and values close to 0 indicate failed segmentation. However, as using only one validation measure can cause bias to our conclusions, we calculated also Dice scores and precision and recall between the automatic and manually drawn segments at validation phase. Following the notation from the definition of the Jaccard index, Dice score is calculated as

2 $$\begin{aligned} Dice(A,B) = \frac{2\cdot |A \cap B|}{|A| + |B|}. \end{aligned}$$

Similarly, precision and recall reflecting the proportion of the overlapping voxels out of all voxels in the automatic and manual segment, respectively, are formally defined as

3 $$\begin{aligned} Precision(A,B)&= \frac{|A \cap B|}{|A|}\end{aligned}$$

4 $$\begin{aligned} Recall(A,B)&= \frac{|A \cap B|}{|B|}, \end{aligned}$$

where the notation again follows the definition of the Jaccard index.

At the beginning of the preprocessing tests, we compared classical denoising approaches to see if some of them are better suited for processing PET images prior to segmentation than others. The used approaches were Wavelet denoising (wavelet), total variation denoising (tv), non-local means denoising (nlmeans), median filtering (median), and Gaussian filtering (gaussian). These methods’ implementations provided in python packages scikit-image (version 0.19.3), dipy (version 1.6.0), and scipy (version 1.9.1) were used. Hatt et al. claim Gaussian filtering to improve segmentation of PET images in case of 3D images containing tumours [24]. In addition to the classic approaches listed here, we considered two sophisticated and rather new denoising methods ‘Block-matching and 3D filtering’ (BM4D) [25] and similarity filtering [26]. However, BM4D took longer than 2 h to process one image and the code for similarity filtering is not publicly available at the time of writing, so we evaluated these two methods only very briefly using two images from each rat dataset. The authors of similarity filtering kindly provided us the filtered data used for clustering in this study. Section 3 of Supplementary text offers more details about the usage of these two methods.

The second evaluated preprocessing type was scaling. Besides *z*-score and logistic scaling, we wanted to test how scaling the TACs so that the clustering would be done based on TACs’ shape rather than their total intensities would affect the clustering accuracies of different methods. Thus, we divided each voxel’s intensity at each time point with its sum over time to obtain the scaled voxel intensities $\overset{*}{v}$. The scaling for voxel *v* at time point *i* is

5 $$\begin{aligned} \overset{*}{v^i}\ = \frac{v^i}{\sum \limits _{t=1}^{T} v^t}, \end{aligned}$$

where *T* is the total number of time points in the dynamic scan. Similar scaling for continuous intensity function defined with kinetic parameters has been used for segmenting dynamic PET images at [10].

**Fig. 2:**
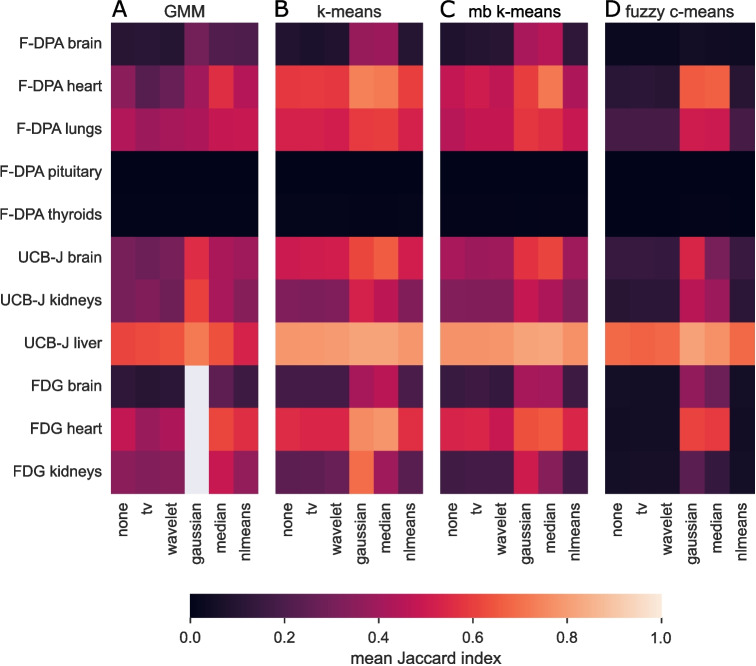
Mean Jaccard indices over images for **A** GMM, **B** *k*-means, **C** mini-batch *k*-means, and **D** fuzzy *c*-means. In all subfigures rows correspond to VOIs, columns indicate the tested denoising approaches, and the cell colour reflects the mean Jaccard index. Notably, GMM did not run with any Gaussian filtered FDG images; thus, mean Jaccard indices cannot be calculated from them

After selecting the best denoising approach and scaling, we evaluated if dimensionality reduction would further improve the results. We tested following dimensionality reduction approaches: truncated singular value decomposition (t-SVD), principal component analysis (PCA), independent component analysis (ICA), and pattern PCA (p-PCA). We implemented the p-PCA to investigate if incorporating spatial information by using a summary of each voxels’ neighbourhood intensities over time would improve the results. It combines principal components of the immediate neighbourhood of each voxel by concatenating them in fixed order. The code implementing p-PCA is available at https://github.com/rklen/Preprocessing_and_Segmentation_Evaluation_PET. Technically, ICA is not a dimensionality reduction method at all, but it can be used as one so it is included here. The utilised Python package was version 1.1.2 of scikit-learn, and the default number of components to extract was 30, but in Supplementary text, we evaluated how using 5–45 principal components affects the results. As p-PCA concatenates the principal components of a neighbourhood (four per adjacent voxel), it provided 27*4=108 principal components instead of the default 30. As pattern PCA provides larger input than the other dimensionality reduction methods, also, clustering times are longer with it. Notably, we also considered dimensionality reduction approaches factor analysis, kernel PCA, t-distributed stochastic neighbour embedding, multidimensional scaling, and isometric mapping, but excluded them due to memory errors.

We wanted to evaluate if segmentation methods not usable for the large images would perform well if the computational issues could be solved. Thus, we tested different methods, including previously unusable ones ‘Balanced Iterative Reducing and Clustering using Hierarchies’ (BIRCH), mean shift, DBSCAN, spectral clustering, slic, watershed, and random walker, on small subimages. For this purpose, we extracted five voxels thick part from all F-DPA data images. The location was chosen so that the pituitary gland, which is a difficult VOI to segment, fitted within the selected area. For clustering methods, we used images preprocessed with denoising, scaling, and dimensionality reduction, but segmentation methods slic, watershed, and random walker expect more conventional images. For them, we used denoised images with all time points summed together. We did not fine-tune the available parameters (if any), but briefly tested different compactness values from logarithmic scale for slic, as recommended in its user instructions. The best mean Jaccard index for the pituitary gland was achieved with compactness level of 0.001, and the results with this parameter value are reported in this manuscript. Random walker has parameter beta, but altering it did not improve the results, so we used the default value of 130.

## Results

### Denoising and Filtering

We tested five classical and relatively fast denoising approaches combined with the four fastest clustering methods. Our results show that Gaussian filtering and median filtering outperform the other denoising approaches and their effect is considerable as compared to segmenting raw images (Fig. 2). Particularly, segmentation with fuzzy *c*-means benefited from denoising with these two approaches (Fig. 2D). The visualised mean Jaccard indices are available in numeric format at Table S2A in page 12 of Supplementary text. The smallest VOIs, namely the pituitary glands and the thyroid glands in F-DPA data, were the hardest ones to segment and denoising did not improve their Jaccard indices. Notably, GMM failed to run when applied on about half of F-DPA images, half of UCB-J images, and all FDG images if Gaussian filtering was used. Despite that, it achieved its highest mean Jaccards with Gaussian filtering in 5 out of 8 VOIs. Particularly, the kidneys benefited from Gaussian filtering as it provided the highest Jaccard indices for all methods in both UCB-J and FDG data.

To choose between median filtering and Gaussian filtering, we compared the obtained Jaccard indices with the Wilcoxon signed-rank test. For *k*-means and mini-batch *k*-means, the differences were minor (*p*-values 0.890, and 0.937, respectively), for fuzzy *c*-means Gaussian filtering was significantly better (*p*-value < 0.001), and for GMM Gaussian filtering provided higher mean Jaccard index than median filtering on 5 out of 8 VOIs, though the difference was not statistically significant (*p*-value 0.098). As shown in Section 4 of Supplementary text, we also tested that the performance difference between Gaussian filtering and median filtering is not due to different kernel sizes. Thus, we continued our analyses with Gaussian filtered data. Guo et al. have suggested that removing the first time points as they tend to be particularly noisy benefits the automatic segmentation of smaller 3D PET images [14]. Our results do not support the claim on modern 4D PET images (Section 5 in Supplementary text), and thus, the remaining analyses were done on images with all measured time points. In Section 3 of Supplementary text, we also show with few example images that two new and sophisticated, but slow or restricted access denoising methods BM4D [25, 27] and similarity filtering [26] provide strong results, though Gaussian filtering still outperforms them with other methods than GMM. Section 3 of Supplementary text also provides visualisation of an example image after preprocessing with each denoising method. Particularly, similarity filtering provided intuitive results suited for visual inspection.

### Scaling and Dimensionality Reduction

**Fig. 3:**
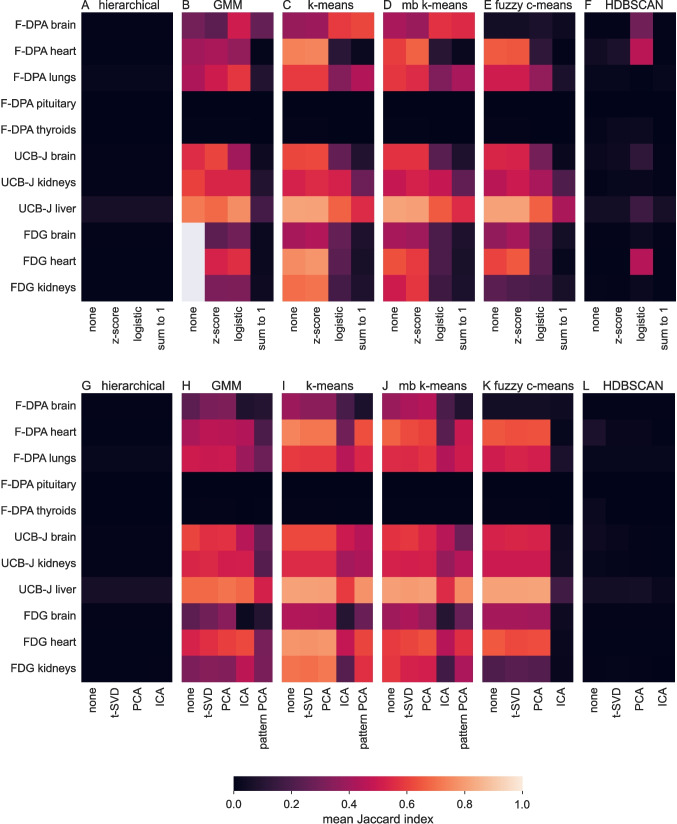
Mean Jaccard indices (indicated by the colour) of each VOI and segmentation method for **A**–**F** different scalings and **G**–**L** dimensionality reduction approaches. In **A**–**F**, the column labelled as ‘none’ refers to denoised data without any scaling, and in **G**–**L**, it denotes denoised and scaled data without any dimensionality reduction (i.e. corresponds to column ‘*z*-score’ in **A**–**F**). Pattern PCA is missing from **G**, **K**, and **L** because it made the corresponding clustering methods very slow (over 2 h per image)

Among the three tested scalings, logistic scaling and especially sum-to-1 scaling drastically decreased the Jaccard indices of *k*-means, mini-batch *k*-means, and fuzzy *c*-means results while *z*-scoring had small, but consistent positive effect on them (Fig. 3A–F, Table S2B in Supplementary text). However, with *k*-means and mini-batch *k*-means, there was one interesting exception to the negative effect of sum-to-1 scaling: the segmentation for the brain from F-DPA data improved (Fig. 3C, D). The brain from F-DPA data is rather difficult VOI to segment as it has low tracer uptake (Section 1 of Supplementary text). For GMM, none of the tested scaling approaches had systematically superior performance, but sum-to-1 was clearly inferior to the other options. Notably, when any scaling was used, GMM had stable performance and it did not throw errors with any of the analysed images. None of the scalings improved the results from hierarchical clustering or HDBSCAN to the level of the other methods, but HDBSCAN systematically benefited from logistic scaling (Fig. 3F). We used *z*-scored data for the remaining analyses.

PCA and t-SVD outperformed ICA and pattern PCA in most VOIs with all clustering methods (Fig. 3G–L, Table S2C in Supplementary text). There were two exceptions to this as GMM identified the heart and the kidneys from FDG data particularly well if ICA was used prior to clustering (Fig. 3H). On the other hand, pattern PCA increased the running times of the clustering methods without any positive impact on the results. While the results from PCA and t-SVD were very similar and there was no statistically significant difference between them (*p*-value > 0.1 in Wilcoxon signed-rank test for each clustering method), we continue our analyses with PCA as it has been successfully used in segmenting smaller PET images [10].

Notably, our results indicate that denoising is the most important preprocessing step, as it improved the mean Jaccards over 50% for all tested methods. Scaling without other preprocessing steps benefitted mainly *k*-means and mini-batch *k*-mean results by increasing their mean Jaccard indices by 14% and 16%, respectively. Dimensionality reduction alone did not systematically improve the results, though it is useful for running time (Table S2 in Section 7 of the Supplementary text). If dimensionality reduction is used, scaling the denoised data is recommended as it improves particularly *k*-means results (Fig. S10 in Section 7 of the Supplementary text). We also tested how the number of utilised principal components affects the Jaccard indices. Our results suggest that using 4–6 of them improves the results and reduces the running times; thus, we used five principal components in the remaining analyses in this study (Section 8 of Supplementary text).

**Fig. 4:**
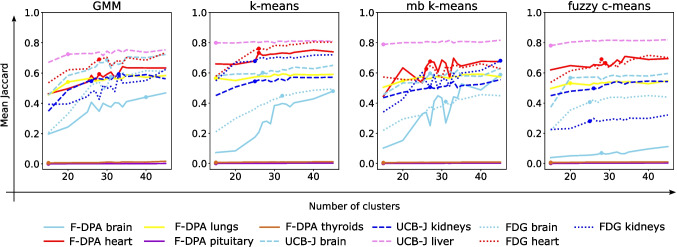
Mean Jaccard indices indicated as a function of cluster number for different methods and VOIs. Every five cluster numbers from interval 15–45 were initially tested, and as interval 25–35 was the most promising, each number from it was added to our tests. This explains the unstable behaviour around 30 clusters present in the figure. A plateau point after which increasing the number of clusters do not increase the mean Jaccard more than 0.05 is marked to each curve

### Number of Clusters

Next, we evaluated how many clusters should be used at the actual segmentation step. As hierarchical clustering and HDBSCAN are slow to run and systematically provided the weakest results with all tested pre-processings, they are excluded from further analyses. We tested every five number of clusters from interval 15–45, and as our initial choice of 30 clusters turned out to be a decent guess, we evaluated every cluster number from interval 25–35. Notably, as Jaccard index is calculated using the combination of one or more clusters with the best match to the VOI, high number of small clusters is expect to increase the Jaccard indices in general. However, increasing the number of clusters increase the running times too, so we aim to identify smallest number of clusters providing results close to the optimal. For each VOI, we calculated a plateau point, after which the mean Jaccard index does not increase more than 0.05 units even if the number of clusters is increased within our tested range.

**Table 3:** Median Jaccard indices, Dice scores, precision, and recall values with the corresponding standard deviations indicated in the parenthesis for all VOIs (rows) and methods (columns) over the validation images

Jaccard	GMM	*k*-means	mb *k*-means	Fuzzy *c*-means
F-DPA brain	**0**.**31** (0.13)	0.27 (0.15)	0.28 (0.14)	0.06 (0.04)
F-DPA heart	0.58 (0.13)	*0.72* (0.15)	0.62 (0.18)	0.70 (0.14)
F-DPA lungs	0.58 (0.10)	0.62 (0.10)	*0.64* (0.09)	0.57 (0.10)
F-DPA pituitary	0.00 (0.00)	0.00 (0.00)	0.00 (0.00)	*0.00* (0.00)
F-DPA thyroids	0.01 (0.01)	*0.01* (0.01)	0.01 (0.01)	0.01 (0.01)
UCB-J brain	**0**.**67** (0.12)	0.58 (0.11)	0.57 (0.12)	0.55 (0.10)
UCB-J kidneys	0.47 (0.16)	*0.48* (0.14)	0.43 (0.16)	0.43 (0.16)
UCB-J liver	0.75 (0.05)	0.80 (0.04)	0.80 (0.05)	**0**.**81** (0.04)
FDG brain	**0**.**54** (0.09)	0.37 (0.05)	0.34 (0.07)	0.38 (0.05)
FDG heart	0.67 (0.10)	**0**.**71** (0.08)	0.62 (0.10)	0.60 (0.09)
FDG kidneys	0.60 (0.20)	**0**.**72** (0.14)	0.66 (0.22)	0.20 (0.26)
Dice	GMM	*k*-means	mb *k*-means	Fuzzy *c*-means
F-DPA brain	*0.48 (0.15)*	0.43 (0.21)	0.44 (0.19)	0.10 (0.07)
F-DPA heart	0.73 (0.12)	*0.84* (0.13)	0.77 (0.16)	0.82 (0.12)
F-DPA lungs	0.73 (0.09)	0.77 (0.07)	*0.78* (0.07)	0.73 (0.08)
F-DPA pituitary	0.00 (0.00)	0.00 (0.00)	0.00 (0.00)	*0.00* (0.00)
F-DPA thyroids	0.01 (0.02)	*0.01* (0.01)	0.01 (0.01)	0.01 (0.01)
UCB-J brain	**0**.**80** (0.09)	0.74 (0.10)	0.73 (0.10)	0.71 (0.09)
UCB-J kidneys	0.64 (0.17)	*0.64* (0.16)	0.60 (0.17)	0.60 (0.19)
UCB-J liver	0.86 (0.04)	0.89 (0.02)	0.89 (0.03)	**0**.**90** (0.03)
FDG brain	**0**.**70** (0.09)	0.54 (0.05)	0.51 (0.08)	0.55 (0.05)
FDG heart	0.80 (0.07)	**0**.**83** (0.05)	0.77 (0.08)	0.75 (0.07)
FDG kidneys	0.75 (0.19)	**0**.**83** (0.13)	0.79 (0.23)	0.33 (0.27)
Precision	GMM	*k*-means	mb *k*-means	Fuzzy *c*-means
F-DPA brain	*0.34* (0.14)	0.31 (0.16)	0.33 (0.16)	0.06 (0.04)
F-DPA heart	0.65 (0.20)	0.84 (0.16)	0.72 (0.22)	*0.86* (0.17)
F-DPA lungs	0.76 (0.19)	0.81 (0.15)	*0.82* (0.14)	0.76 (0.16)
F-DPA pituitary	0.00 (0.00)	0.00 (0.00)	0.00 (0.00)	**0**.**00** (0.00)
F-DPA thyroids	0.01 (0.01)	*0.01* (0.01)	0.01 (0.01)	0.01 (0.01)
UCB-J brain	0.70 (0.13)	*0.71* (0.14)	0.66 (0.13)	0.66 (0.15)
UCB-J kidneys	0.62 (0.23)	**0**.**76** (0.24)	0.63 (0.23)	0.60 (0.22)
UCB-J liver	0.85 (0.07)	*0.92* (0.04)	0.91 (0.04)	0.90 (0.04)
FDG brain	**0**.**62** (0.16)	0.43 (0.07)	0.42 (0.10)	0.47 (0.07)
FDG heart	0.72 (0.13)	*0.85* (0.10)	0.70 (0.10)	0.66 (0.12)
FDG kidneys	0.71 (0.28)	**0**.**91** (0.16)	0.84 (0.29)	0.22 (0.36)
Recall	GMM	*k*-means	mb *k*-means	Fuzzy *c*-means
F-DPA brain	**0**.**92** (0.14)	0.79 (0.22)	0.74 (0.19)	0.49 (0.18)
F-DPA heart	0.88 (0.11)	0.86 (0.15)	*0.90* (0.14)	0.77 (0.15)
F-DPA lungs	0.92 (0.07)	0.94 (0.06)	0.93 (0.06)	*0.95* (0.07)
F-DPA pituitary	0.61 (0.29)	*0.73* (0.21)	0.63 (0.21)	0.61 (0.20)
F-DPA thyroids	*0.84* (0.12)	0.76 (0.10)	0.79 (0.12)	0.69 (0.12)
UCB-J brain	**0**.**95** (0.14)	0.75 (0.11)	0.75 (0.12)	0.72 (0.13)
UCB-J kidneys	**0**.**90** (0.09)	0.84 (0.09)	0.86 (0.08)	0.83 (0.10)
UCB-J liver	0.88 (0.07)	0.86 (0.04)	0.88 (0.06)	*0.89* (0.04)
FDG brain	**0**.**81** (0.09)	0.74 (0.10)	0.69 (0.12)	0.66 (0.09)
FDG heart	0.89 (0.11)	0.88 (0.10)	*0.93* (0.12)	0.91 (0.13)
FDG kidneys	*0.85* (0.12)	0.82 (0.08)	0.76 (0.14)	0.74 (0.17)

If a median is significantly higher than obtained with any other method (Table 4), it is **bolded**. Otherwise, the highest median is highlighted with *italic*. One rat from the F-DPA dataset was oddly positioned in the scanner, and the brain and the pituitary gland were not within the scanned area; thus, those VOIs are missing from the reported medians and standard deviations

**Table 4:** *p*-values indicating the significance level of differences between Jaccard indices, Dice scores, precision, and recall values obtained with different methods (columns) from different VOIs (rows)

Jaccard	GMM vs k-m	GMM vs mb	GMM vs fuzzy	k-m vs mb	k-m vs fuzzy	mb vs fuzzy
F-DPA brain	0.011	0.034	0.000	0.300	0.000	0.000
F-DPA heart	0.000	0.237	0.002	0.006	0.111	0.033
F-DPA lungs	0.002	0.001	0.227	0.408	0.012	0.001
F-DPA pituitary	0.672	0.751	0.003	0.711	0.005	0.034
F-DPA thyroids	0.617	0.123	0.280	0.053	0.043	0.653
UCB-J brain	0.000	0.000	0.000	0.018	0.000	0.465
UCB-J kidneys	0.124	0.002	0.000	0.008	0.000	0.062
UCB-J liver	0.000	0.000	0.000	0.705	0.045	0.032
FDG brain	0.000	0.000	0.000	0.157	0.568	0.026
FDG heart	0.032	0.152	0.003	0.000	0.000	0.125
FDG kidneys	0.000	0.492	0.000	0.000	0.000	0.000
Dice	GMM vs k-m	GMM vs mb	GMM vs fuzzy	k-m vs mb	k-m vs fuzzy	mb vs fuzzy
F-DPA brain	0.008	0.034	0.000	0.263	0.000	0.000
F-DPA heart	0.001	0.380	0.003	0.006	0.116	0.033
F-DPA lungs	0.002	0.001	0.247	0.380	0.007	0.001
F-DPA pituitary	0.672	0.751	0.003	0.711	0.005	0.034
F-DPA thyroids	0.617	0.123	0.280	0.049	0.043	0.635
UCB-J brain	0.000	0.000	0.000	0.023	0.000	0.477
UCB-J kidneys	0.134	0.003	0.000	0.009	0.000	0.060
UCB-J liver	0.000	0.000	0.000	0.678	0.041	0.028
FDG brain	0.000	0.000	0.000	0.141	0.636	0.025
FDG heart	0.027	0.157	0.003	0.000	0.000	0.130
FDG kidneys	0.000	0.595	0.000	0.000	0.000	0.000
Precision	GMM vs k-m	GMM vs mb	GMM vs fuzzy	k-m vs mb	k-m vs fuzzy	mb vs fuzzy
F-DPA brain	0.012	0.071	0.000	0.164	0.000	0.000
F-DPA heart	0.001	0.483	0.000	0.014	0.053	0.000
F-DPA lungs	0.582	0.258	0.745	0.901	0.452	0.143
F-DPA pituitary	0.692	0.751	0.002	0.711	0.005	0.034
F-DPA thyroids	0.008	0.708	0.084	0.004	0.582	0.002
UCB-J brain	0.172	0.018	0.045	0.217	0.421	0.733
UCB-J kidneys	0.003	0.832	0.379	0.005	0.000	0.217
UCB-J liver	0.000	0.000	0.000	0.561	0.203	0.512
FDG brain	0.000	0.000	0.000	0.608	0.001	0.003
FDG heart	0.063	0.087	0.025	0.000	0.000	0.839
FDG kidneys	0.000	0.146	0.000	0.003	0.000	0.000
Recall	GMM vs k-m	GMM vs mb	GMM vs fuzzy	k-m vs mb	k-m vs fuzzy	mb vs fuzzy
F-DPA brain	0.000	0.000	0.000	0.034	0.000	0.000
F-DPA heart	0.208	0.980	0.024	0.089	0.007	0.001
F-DPA lungs	0.199	0.367	0.063	0.901	0.671	0.565
F-DPA pituitary	0.236	0.563	0.864	0.304	0.037	0.291
F-DPA thyroids	0.006	0.361	0.003	0.040	0.025	0.001
UCB-J brain	0.000	0.000	0.000	0.160	0.071	0.803
UCB-J kidneys	0.000	0.000	0.000	0.057	0.890	0.068
UCB-J liver	0.410	0.890	0.612	0.665	0.027	0.139
FDG brain	0.000	0.000	0.000	0.107	0.000	0.076
FDG heart	0.706	0.272	0.854	0.189	0.750	0.182
FDG kidneys	0.272	0.008	0.001	0.058	0.017	0.608

The *p*-values are rounded to three decimals. Abbreviations $k-m$, *mb*, and *fuzzy* refer to *k*-means, mini-batch *k*-means, and fuzzy *c*-means, respectively

While the trend differences between the methods were minor, the cluster number has weaker effect on the performance of fuzzy *c*-means as compared to the other methods (Fig. 4). Different tracers did not systematically benefit from different cluster numbers either (Section 8 of Supplementary text). Increasing the number of clusters increased the mean Jaccard indices for most VOIs, and the phenomenon was particularly strong for the brain. On the other hand, all methods’ performances for the liver from UCB-J data and the lungs from F-DPA data stabilised already with 15 or 20 clusters. For further analyses, we use median plateau point over the VOIs (excluding the two smallest VOIs in F-DPA data that are never detected) for each clustering method. This claimed cluster numbers 28, 26, 27, and 25 for GMM, *k*-means, mini-batch *k*-means, and fuzzy *c*-means, respectively.

### Results with Validation Images

To confirm that the obtained accuracies were not a coincidence, we run the best-performing clustering methods for the remaining validation images preprocessed with the observed best practices. The results are similar to those obtained with the 10 test images from each dataset. Overall, the liver from the UCB-J data was the easiest VOI to segment, but the results were also good for the heart from the F-DPA and FDG data and for the kidneys from the FDG data (Table 3). *K*-means had the highest median precision in six out of eleven organs, whereas GMM outperformed the other methods in six organs according to recall values. Despite outperforming the other methods at segmenting the liver from the UCB-J data, fuzzy *c*-means had the least reliable performance as the other methods had over three-fold better median Jaccard index for the brain in the F-DPA data and for the kidneys in the FDG data (Table 3). In both of these examples, precision and recall of the fuzzy *c*-means are clearly inferior to the other methods, precision being the biggest issue.

GMM significantly outperformed the other methods in segmenting brain from all datasets (Table 4), yet *k*-means and mini-batch *k*-means had the most stable performance over different VOIs as they did not have the lowest median Jaccard with *p*-value $<0.05$ in any VOI. *K*-means also significantly outperformed the other methods at segmenting the heart and the kidneys from the FDG data (Table 4).

**Fig. 5:**
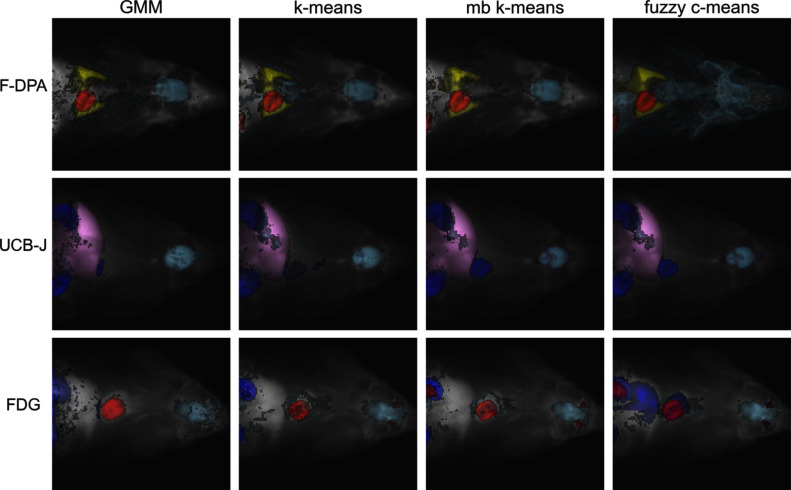
Clusters representing different VOIs for different methods (columns) and datasets (rows). The image representing each tracer is selected so that the Jaccard indices were within one standard deviation from the median for all VOIs and methods. If multiple images filled this criterion, the image was selected randomly among the candidates. The pituitary gland is excluded from the F-DPA visualisation, as it was part of a big cluster making the figure difficult to read

Notably, often, the heart and the kidneys clustered partially together, particularly in the FDG and UCB-J data (Fig. 5, Section 9 of Supplementary text). In the F-DPA example image in Fig. 5, the thyroid glands also clustered together with the heart, but this was not a common phenomenon among the images (see Section 9 of Supplementary text). Whereas in the FDG data, the heart and the Harderian glands typically clustered together (Fig. 5, Section 9 of Supplementary text) with other methods than GMM.

**Fig. 6:**
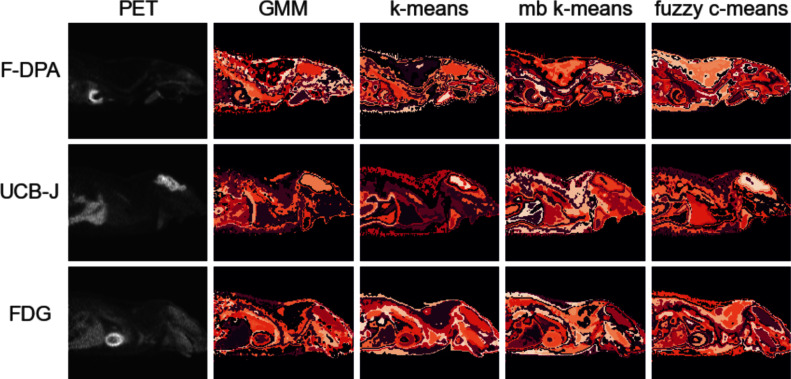
Full segmentation of the middle slice (sagittal view) for one example image from each tracer (rows) provided by different methods (columns). The example images are the same ones than in the Fig. 5. The corresponding slice of the original PET image on the left-most column is given for reference

There is no distinct difference in the output clusters of the four most promising methods, but they all generate remotely equally sized clusters that tend to form one or more capsule clusters around high intensity areas (Fig. 6). Notably, the slower methods with lower Jaccard indices, hierarchical clustering and HDBSCAN, behave drastically differently (Fig. S14 in Section 10 of Supplementary text).

To evaluate the performance of wider range of methods, if they were computationally usable, we tested the segmentation methods on small images (Section 10 of Supplementary text). With the reduced data, also, clustering methods BIRCH, mean shift, DBSCAN, and spectral clustering, as well as region-based methods slic, watershed, and random walker were usable. The results show that among the computationally demanding methods particularly spectral clustering and slic have potential. Another important observation from those tests is the improved, but still insufficient, segmentation of small VOI pituitary gland.

**Fig. 7:**
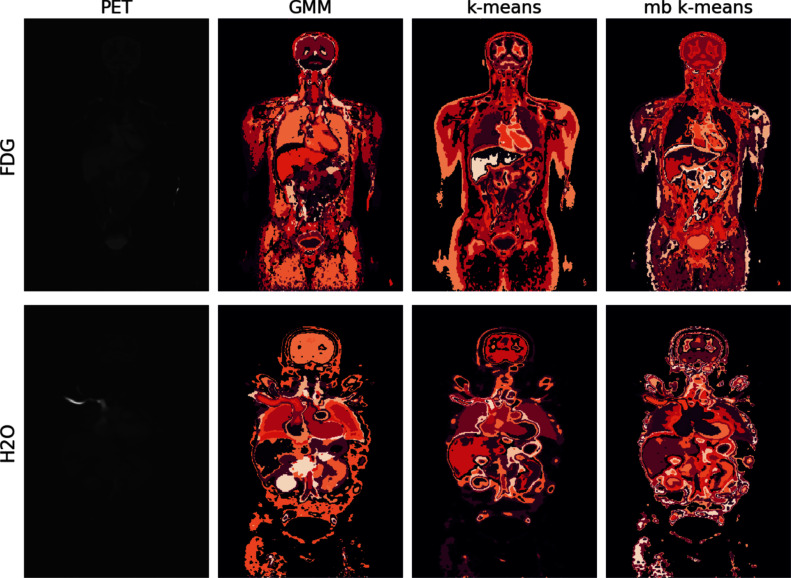
Segmentations of the middle slice of a randomly selected image from the FDG human data (top row) and the H2O human data (bottom row). The left-most panel visualises the corresponding slice of the PET image prior to preprocessing (sum over time points)

**Table 5:** Median Jaccard indices, Dice scores, precision, and recall values with the corresponding standard deviations indicated in the parenthesis for different methods

	Median (standard deviation)			*p*-value		
Jaccard	GMM	*k*-means	mb *k*-means	GMM vs k-m	GMM vs mb	k-m vs mb
Brain	**0**.**77** (0.04)	0.74 (0.04)	0.74 (0.04)	0.00	0.00	0.22
Aorta	0.19 (0.04)	*0.20* (0.03)	0.20 (0.03)	0.10	0.08	0.52
Kidneys	**0**.**63** (0.04)	0.43 (0.13)	0.48 (0.09)	0.00	0.00	0.19
Liver	**0**.**78** (0.05)	0.74 (0.06)	0.73 (0.06)	0.00	0.00	0.05
Lungs	**0**.**74** (0.05)	0.60 (0.09)	0.67 (0.07)	0.00	0.00	0.00
Heart	**0**.**56** (0.05)	0.52 (0.06)	0.51 (0.04)	0.00	0.00	0.77
Dice	GMM	*k*-means	mb *k*-means	GMM vs k-m	GMM vs mb	k-m vs mb
Brain	**0**.**87** (0.03)	0.85 (0.03)	0.85 (0.03)	0.00	0.00	0.23
Aorta	0.33 (0.06)	*0.33* (0.05)	0.33 (0.04)	0.10	0.10	0.50
Kidneys	**0**.**77** (0.03)	0.61 (0.13)	0.65 (0.09)	0.00	0.00	0.15
Liver	**0**.**88** (0.03)	0.85 (0.04)	0.84 (0.04)	0.00	0.00	0.04
Lungs	**0**.**85** (0.03)	0.75 (0.07)	0.80 (0.05)	0.00	0.00	0.00
Heart	**0**.**72** (0.04)	0.68 (0.06)	0.68 (0.04)	0.00	0.00	0.75
Precision	GMM	*k*-means	mb *k*-means	GMM vs k-m	GMM vs mb	k-m vs mb
Brain	*0.85* (0.04)	0.83 (0.04)	0.81 (0.04)	0.10	0.03	0.63
Aorta	*0.21* (0.05)	0.21 (0.04)	0.21 (0.04)	0.04	0.05	0.82
Kidneys	*0.84* (0.09)	0.76 (0.21)	0.75 (0.16)	0.08	0.01	0.68
Liver	**0**.**92** (0.05)	0.84 (0.06)	0.85 (0.06)	0.00	0.01	1.00
Lungs	**0**.**91** (0.03)	0.78 (0.12)	0.84 (0.06)	0.00	0.02	0.00
Heart	*0.71* (0.05)	0.67 (0.05)	0.66 (0.06)	0.24	0.10	0.50
Recall	GMM	*k*-means	mb *k*-means	GMM vs k-m	GMM vs mb	k-m vs mb
Brain	**0**.**91** (0.05)	0.88 (0.05)	0.89 (0.05)	0.04	0.00	0.92
Aorta	*0.83* (0.17)	0.76 (0.10)	0.73 (0.09)	0.58	0.64	0.63
Kidneys	**0**.**74** (0.07)	0.54 (0.13)	0.54 (0.13)	0.01	0.00	0.34
Liver	0.87 (0.05)	*0.87* (0.03)	0.85 (0.04)	0.76	0.37	0.14
Lungs	*0.77* (0.07)	0.77 (0.07)	0.73 (0.07)	0.37	0.01	0.15
Heart	*0.80* (0.07)	0.70 (0.11)	0.70 (0.04)	0.05	0.02	0.54

The highest median is **bolded** if the method’s scores (e.g. Jaccard indices) are significantly higher than the other methods’, as indicated by the *p*-values reported here. Otherwise, the highest median is highlighted by *italic* font

### Human Images

To see if the segmentation and preprocessing algorithms are computationally feasible also with the large human data, we initially applied the four most promising methods to eight preprocessed human images (comprising three [$^{18}$F]FDG and five [$^{15}$O]H$_2$O scans). Notably, fuzzy *c*-means threw memory errors indicating its unsuitability for segmenting full human total-body images. The overall segmentation results are similar to those obtained from the rat data (Figs. 6 and 7). The phenomenon of high intensity areas having a core segment surrounded with one or more layers of capsulating segments was observed already with the rat datasets. In the human data, it is particularly strongly present in mini-batch *k*-means segmentation of the H2O human data (Fig. 7). Section 11 of the Supplementary text provides visualisation of the segmentations of all the analysed human images.

Based on these observations, we applied the three feasible methods (GMM, *k*-means, and mini-batch *k*-means) on 22 human images with manually drawn segments available for more objective validation. Interestingly, in these results on human images, GMM outperforms the other two methods in most cases according to all used evaluation measures (Table 5). GMM had significantly higher median Jaccard index, Dice score, precision, and recall than *k*-means or mini-batch *k*-means in almost all organs. Among the evaluated organs, the aorta was the hardest one to segment due to other areas segmenting together with it, as suggested by high recall, but low precision values (Table 5).

## Discussion and Conclusions

This study aids further method development for segmenting large modern PET images by evaluating different basic tools covering multiple different types of widely used preprocessing and segmentation methods. Multiple datasets with different tracers were used to ensure that the conclusions are not biased to some type of PET images. Using known segments of different sizes, shapes, and intensities serves the same purpose. We also tested several earlier suggestions to enhance segmentation in smaller PET images or restricted areas. Our results suggest that preprocessing considerably improves the segmentation results, denoising being the most important step. Success of the Gaussian filtering as a denoising approach is not surprising as the noise in PET images have been reported to be mainly, but not exclusively, Gaussian noise [28, 29]. Among the tested segmentation methods, *k*-means and particularly GMM appear the most robust choices for further development, but the selection of the suitable number of clusters remains difficult. Excluding very small VOIs, the accuracies obtained here are in line with those of the older methodology designed for smaller images, as mentioned in the introduction. However, comparing results from different studies obtained using different set of images is not recommended.

Despite the improvements preprocessing can bring to the segmentation, small VOIs cannot be segmented with the tested methods that are computationally feasible even for the large dynamic total-body images. There are several ways to tackle the issue: (1) postprocessing the results by for example using connected component analysis, (2) using multiple different segmentation methods and combining the results, (3) dividing the images into smaller pieces and segmenting them independently, or (4) iteratively clustering the image first into coarse segments that are further broken into smaller pieces independently from each other. Guo et al. have suggested computationally light pre-clustering followed by more sophisticated, but intensive second round of clustering in context of segmenting brain regions [30].

The 2-h cutoff for running time used in this study is overly generous for clinical applications, as medical doctors often have very limited time for image analysis. However, the used computer was a regular office desktop and the implementations of different clustering methods were not optimised for efficiency. Using a dedicated powerful analysis computer can speed up the analyses significantly. Using graphic processing unit (GPU) computing for segmentation can also speed up the process more than ten-fold [31, 32]. Notably, using GPU computing would require the implementation of the methods to support it, which is not the case here. Also field-programmable gate array (FPGA) hardware has been shown to double the running speed of *k*-means clustering in the literature [33]. Even without access to GPU or FGPA computing, other hardware improvements, such as efficient multi-thread implementation, have been reported to improve the running times [34]. Thus, the running speed can be considerably improved with different hardware solutions, but the implementation of the selected algorithm needs to support the chosen hardware to get the benefit.

The possibility to combine deep learning with general-purpose unsupervised segmentation is a tempting opportunity to obtain the best of both worlds, namely fast and accurate segmentation of any kind of PET image without heavy requirement for the used hardware. This has already been successfully applied to classify prostate cancer form PET/MRI images [35]. Also, combining deep learning on CT images with unsupervised segmentation of PET images could provide segments that are meaningful both anatomically and functionally. Good supervised segmentation methods to segment many diverse tissues and organs from CT images are already available [36, 37].

All the computationally light enough methods tested here were different clustering approaches. The main weakness of clustering as a segmentation tool is its incapability to naturally consider spatial information, which is important in this application. Thus, either combining clustering with some region-based approach or giving also voxel coordinates in some format as a clustering input could improve the results. According to our tests with the small images, slic was the most promising candidate among the region-based methods to be paired with clustering. In addition, several means to segment small VOIs suggested above would enforce location in the clustering. Namely, splitting the image into smaller subimages and connected component analysis would force the clustering to segment together only nearby located voxels. As the name implies, connected component analysis would provide strictly connected segments, whereas splitting the image would allow non-adjacent voxels to segment together as long as they are within the same sub-image.

PCA has been criticised for capturing mainly filling of bladder from total-body images [15]. The rat images utilised in this study covered the area from pelvis upwards and the bladder did not fit onto the scanned area. However, the human images did include bladder, yet we did not observe any issues related to it despite our preprocessing pipeline including PCA as one step. One potential explanation is the scanning time being 40 min in the FDG human images and less than 5 min in the H2O human images. Longer scanning could generate more issues. The topic needs further research. Other suggestion from the literature unsupported by our results is the benefits of excluding the first time point due to its particularly high noise level [14]. Our results did not systematically benefit from excluding either 1, 3, or 6 first time points. It is possible that the scanners have improved since the statement was published in 2003, or the denoising step in our study improved the data quality so that the first time point is more informative than noisy.

While HDBSCAN results were not very accurate, we were able to run it without problems. This is surprising as our attempts to run DBSCAN caused the computer to crash, yet HDBSCAN is based on DBSCAN. We suspect that the parameter range HDBSCAN automatically investigates is unsuitable for our test data, and causes the runs to be computationally feasible, though most of the voxels are labelled as noise in the results.

This study has several limitations. First of all, the manually segmented clusters serving as gold standard are defined from sum image over time, so the time aspect is not utilised, which wastes information and potentially causes us to miss some interesting distinct subregions. However, manually defining the VOIs from a video was not feasible. In addition, all the manual segmentations were done according to the instructions of a single expert, so subjective bias may occur in them. Also, ideally all combinations of segmentation method, denoising, scaling, and dimensionality reduction would have been tested, but this would have yielded 576 combinations for all three rat datasets and the runs were already very time-consuming with the chosen setup.

As future work, we propose a method that incorporates the identified best pipeline and combines it with (1) some approach to better identify small segments, (2) a way to utilise spatial information, as discussed above, and (3) deep learning-based segmentation of CT images. The last point of combining unsupervised segmentation of PET images with deep learning-based segmentation of CT images could provide segments that are meaningful both anatomically and functionally according to the strengths of the modalities. Adding a supervised method to the segmentation pipeline would also ease the interpretation of the results as the CT segments would have anatomical labels.

Our results provided guidelines for further research, particularly method development for automatically segmenting PET images in unsupervised manner. Based on the observations made here, we believe that the best workflow would include preprocessing (particularly denoising), clustering combined with some approach to consider spatial information and small structures, and suitable postprocessing. Such segmentation pipeline could provide good balance between accuracy, computational usability, and the robustness of unsupervised segmentation. The key conclusions of this study are listed below:

- Most previously used unsupervised segmentation methods do not work with large modern PET images.
- Denoising is the most important preprocessing step.
- GMM and *k*-means are the most promising segmentation method candidates for further method development, GMM having the best performance with human images.
- Small segments cannot be detected with the evaluated approaches.

## Supplementary Information

Below is the link to the electronic supplementary material.

Supplementary file 1 (pdf 3146 KB)

## Data Availability

Our codes, including the implementation of p-PCA are available at https://github.com/rklen/Preprocessing_and_Segmentation_Evaluation_PET. While we are not allowed to share the human data, access to the rat datasets will be added to the same github page https://github.com/rklen/Preprocessing_and_Segmentation_Evaluation_PET once their authors have published them.
