# Supplementary material for: Comparison of Automatic Segmentation and Preprocessing Approaches for Dynamic Total-Body 3D Pet Images with Different Pet Tracers
Source: J Imaging Inform Med. 2025 May 27;39(1):382–99. doi: 10.1007/s10278-025-01540-4 (PMC12920958; doi:10.1007/s10278-025-01540-4)
Supplement: Supplementary file 1 — (pdf 3146 KB) [file 10278_2025_1540_MOESM1_ESM.pdf]

COMPARISON OF AUTOMATIC SEGMENTATION APPROACHES FOR DYNAMIC TOTAL-BODY 3D PET IMAGES WITH DIFFERENT PET TRACERS

SUPPLEMENTARY TEXT

1. Manually segmented VOIs

We manually segmented 5 volumes of interest (VOIs) from F-DPA images, 3 from UCB-J images, and 3 from FDG images. The segmentation was done from PET images using Carimas software (version 2.10) and the segmented VOIs were brain, heart, lungs, pituitary gland, and thyroid glands from the F-DPA data, brain, kidneys, and liver from the UCB-J data, and brain, heart, and kidneys from the FDG data. Figure S1 visualises one example image of the manual segmentation for each tracer.

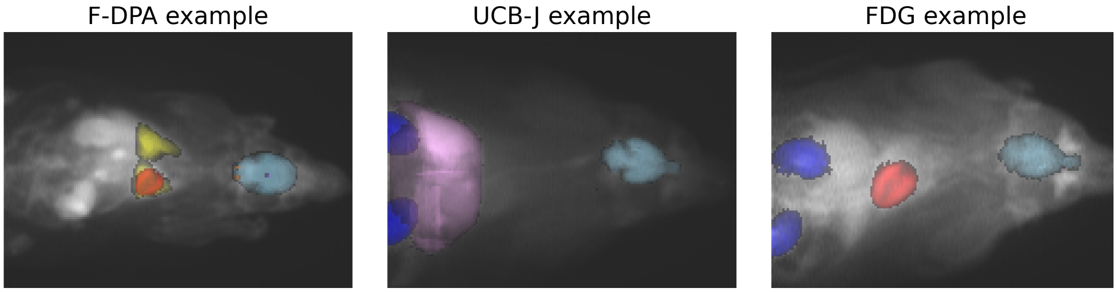

Figure S1. One example image with the manually segmented VOIs from each tracer. The underlying gray-scale image is the PET image (light colour indicates high radio activity over time and hight dimension) and the coloured areas on the top are the manual segments. In the F-DPA example, VOIs thyroid glands and pituitary gland are very small and overlap with the brain in this visualisation.

Liver in UCB-J data was by far the biggest analysed VOI (Figure S2A) and also its tracer intake peaked to the highest level among the VOIs (Figure S2B). Pituitary gland and thyroid glands from the F-DPA data were the smallest VOIs (Figure S2A), and pituitary gland's tracer intake was relatively low but constant, and thyroid glans' radioactivity peak was among the highest ones (Figure S2B). The hearts in the FDG data were bigger than those in the F-DPA data (Figure S2A) because in the FDG data, the whole heart was segmented, whereas from the F-DPA images, only the muscle areas with high tracer

intake were included into the manually segmented VOI. Notably, kidneys did not always fully fit into the scanned area, which explains the high variation in their sizes (Figure S2A). While brain had high radio activity peak in the UCB-J data, its tracer intake was low in the FDG and particularly in the F-DPA data (Figure S2B).

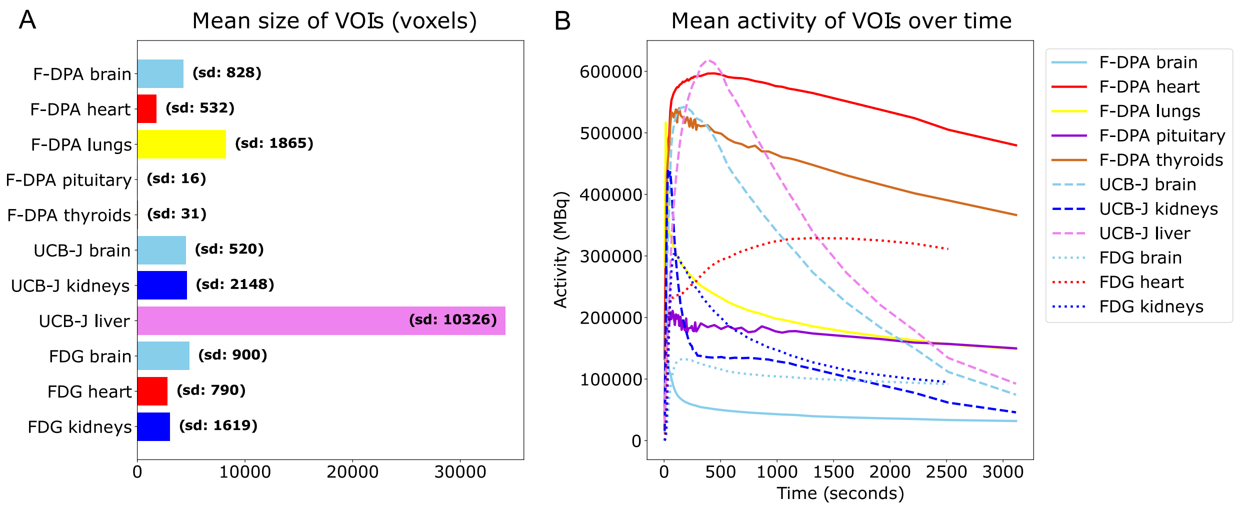

Figure S2. (A) Mean sizes in voxels of all analysed VOIs with the standard deviation indicated in the parenthesis, and (B) mean tracer activity over time (TAC curve) of each analysed VOI. The TACs of the pituitary gland and thyroid glands from the F-DPA data are noisier than those of the other VOIs due to smaller numebr of voxels to contribute to the mean.

2. SUV vs raw data

Here we test if using standardised uptake values (SUVs) instead of raw radioactivities affects the clustering results. In SUV the raw intensity value of a voxel v is scaled with the ratio of decay corrected dose α' and weight w of the scanned body (grams):

$$SUV(v) = \frac{v}{\alpha'/w}. \tag{1}$$

We clustered two images from all three rat datasets based on their raw activity values and SUVs. We evaluated the results by comparing the obtained clusters to manually drawn segments using Jaccard index as a measure of accuracy (see section '2.3. Test design' of the main manuscript). Jaccard indices get values between 0 and 1, where value 1 indicate perfect match between the manual segmentation and the best fitting clusters. We calculated Jaccard indices from segmentation results obtained using SUVs, preprocessed SUVs, intensity values, and preprocessed intensity values. The used preprocessing pipeline included denoising with gaussian filtering, scaling to normal distribution, and dimensionality reduction with principal component analysis.

Our results show that for k-means and fuzzy c-means using SUVs instead of raw activities did not affect the accuracy of the results. For GMM and mini-batch k-means there were bigger variation, but neither datatype was systematically better than the other (Figure S3). As only some of the methods were affected, we believe that the instability of their results in this test is about the methods' sensitivity rather than using raw or standardised input values. Using unprocessed or preprocessed data did not affect difference (or lack of it) between the results obtained from raw intensity values of SUVs.

Thus, we conclude that for clustering based segmentation it is not critical if raw activities or SUVs are used. This is not surprising as SUVs are used to increase the comparability of different images, but clustering is done for images independently from each other.

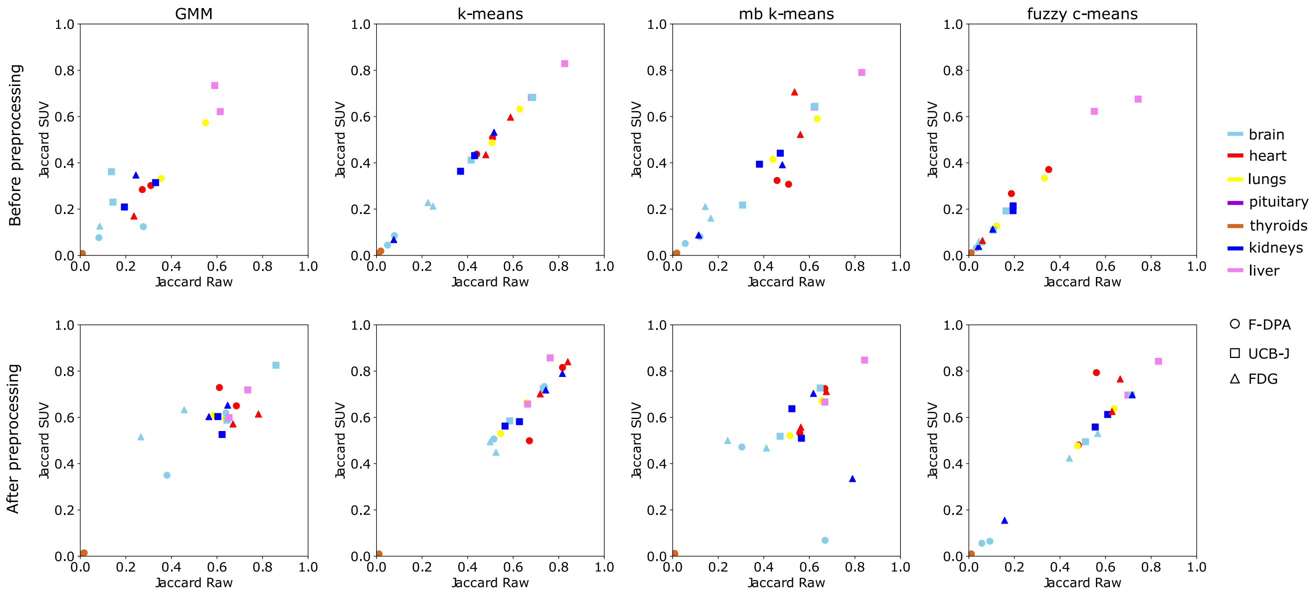

Figure S3. Jaccard indices of different VOIs obtained from intensity values (y-axis) and SUVs (x-axis) using different methods (columns). The clustering was done on the values as such and on preprocessed values (rows).

3. Selecting the number of clusters

We evaluated how many clusters should be used at the actual segmentation step. As hierarchical clustering and HDBSCAN are slow to run and systematically provided the weakest results with all tested pre-processings, they are excluded from further analyses. We tested every five cluster numbers from interval 15-45 and as our initial choice of 30 clusters turned out to be a decent guess, we evaluated every cluster number from interval 25-35. Notably, as Jaccard index is calculated using the combination of one or more clusters with the best match to the VOI, high number of small clusters is expect to increase the Jaccard indices in general. However, increasing the number of clusters increase the running times too, so we aim to identify smallest number of clusters providing results close to the optimal. For each VOI, we calculated a plateau point, after which the mean Jaccard index does not increase more than 0.05 units even if the number of clusters is increased within our tested range.

While the trend differences between the methods were minor, the cluster number has weaker effect on the performance of fuzzy c-means as compared to the other methods (Figure S4). Different tracers did not systematically benefit from different cluster numbers either (Figure S5). Increasing the number of clusters increased the mean Jaccard indices for most VOIs, and the phenomenon was particularly strong for the brain. On the other hand, all methods' performances for the liver from UCB-J data and the lungs from F-DPA data stabilised already with 15 or 20 clusters. For further analyses, we use median

plateau point over the VOIs (excluding the two smallest VOIs in F-DPA data that are never detected) for each clustering method. This claimed cluster numbers 28, 26, 27, and 25 for GMM, k-means, mini-batch k-means, and fuzzy c-means, respectively.

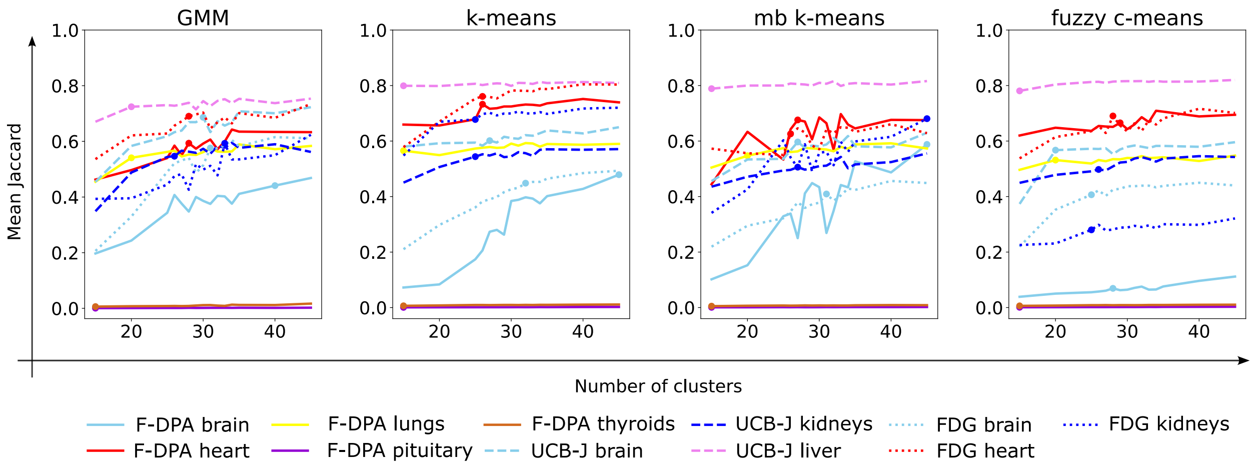

Figure S4. Mean Jaccard indices indicated as a function of cluster number for different methods and VOIs. Every five cluster numbers from interval 15-45 were initially tested and as interval 25-35 was the most promising, each number from it was added to our tests. This explains the unstable behaviour around 30 clusters present in the figure. A plateau point after which increasing the number of clusters do not increase the mean Jaccard more than 0.05 is marked to each curve.

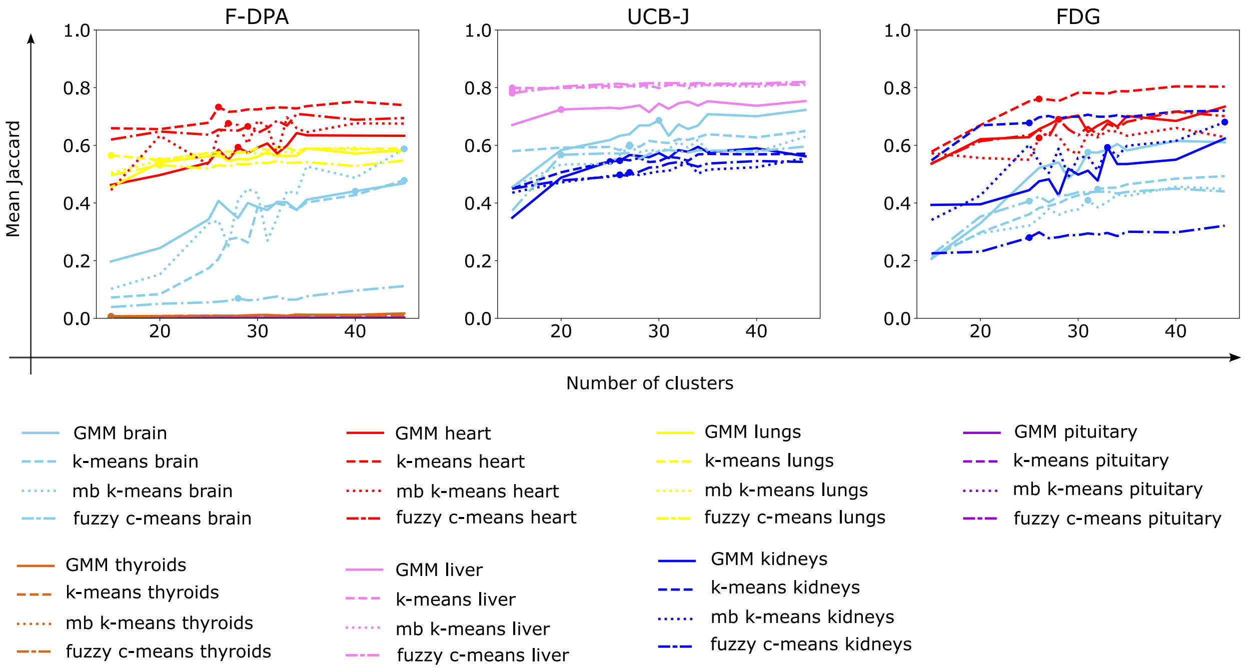

Figure S5. Mean Jaccard indices as a function of cluster number for different tracers and VOIs.

4. Modern, but slow denoising methods

Here we test how suited two novel sophisticated denoising methods are for PET data preprocessing prior to clustering. The two denoising approaches are BM4D [23, 25] and similarity filtering [4]. BM4D provides gentle filtering (Figure S6), though the contrast between the median and maximum activity is less extreme than before denoising (Table S1). Similarity filtering on the other hand heavily alters the overall range of the activity values (Table S1), but provides visually intuitive denoising well suited for human inspection. Figure S6 and Table S1 also show that among the classical denoising methods total variation, wavelet, and non-local means process the data conservatively, whereas 4D kernel based methods gaussian filtering and median filtering drasticly alter the data.

As expected based on Figure S6, results after BM4D are closer to the results from raw data than those from similarity filtered data. Our results show that for most VOIs and segmentation methods BM4D and similarity filtering improve the segmentation as compared to using raw data (Figure S7). Particularly GMM benefits from Similarity filtering. However, despite being unfaithful to the original data (Figure S6), Gaussian filtering typically provided the best mean Jaccard indices for the other methods than GMM.

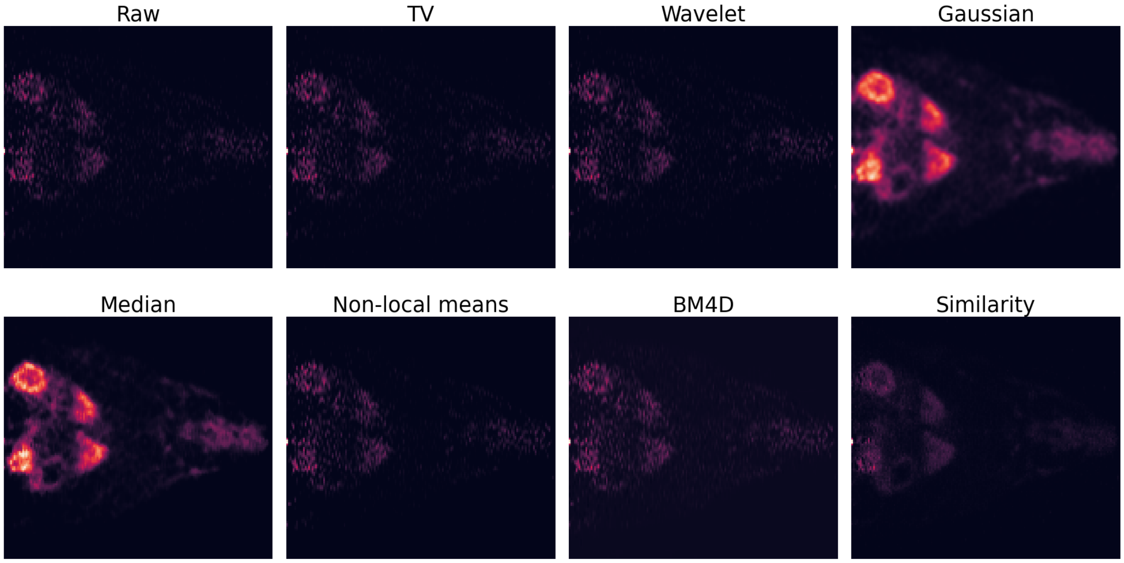

FIGURE S6. The middle slice from coronal view at 6th time point after each denoising algorithm for one example rat from the F-DPA dataset. Notably, the colour range is defined independently for all denosing methods, so the colours are not comparable between the methods.

TABLE S1. Maximum and median intensity values rounded to integer from the slice visualised in Figure S6

	Raw	TV	Wavelet	Gaussian
maximum	3807288	3805683	3807046	795930
median	2052	2560	2111	19105
	Median	Non-local means	BM4D	Similarity
maximum	780066	3807117	3820697	3154
median	2995	0	6697	18

5. Kernel size in median filtering

As the gaussian filtering slightly outperformed median filtering in the main manuscript, we wanted to test if the difference was due to the different kernel sizes (11 for gaussian filtering and 3 for median filtering). Thus, here we test the effect of kernel size to the results from median filtered data. We conclude that the kernel size is not what gave the advantage to the gaussian filtering, as larger kernel had negative or neutral impact on median filtering results in almost all VOIs and segmentation methods (Figure S8). The only instance where larger kernel improved the results was the brain from UCB-J data when the segmentation was done with gmm.

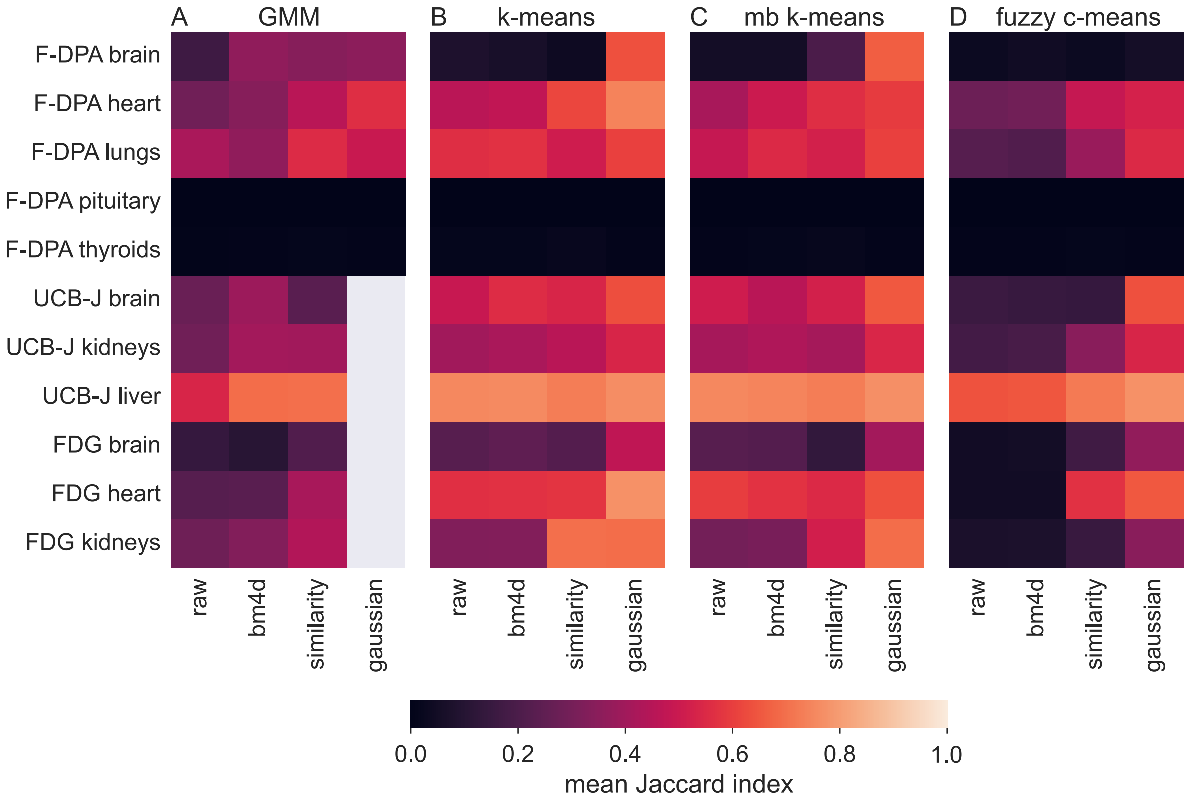

Figure S7. Mean Jaccard indices from raw, bm4d, similarity filtered, and gaussian filtered data for (A) GMM, (B) k-means, (C) mini-batch k-means, and (D) fuzzy c-means. Notably, here the means are calculated over only two images per dataset, so the results for raw data and gaussian filtering are not identical to those illustrated in the main manuscript.

6. The effect of removing the first time points

Guo *et al.* suggested removing first time points from the data prior to segmentation as they are often particularly noisy [28]. Here we tested how removing the first 1, 3, or 6 time points corresponding to the first 10 seconds, 30 seconds, and one minute of the scan affected the Jaccard indices in our 4D datasets. The removal was done after the denoising with gaussian filtering, but before the segmentation.

Our results show that typically the removal had only a minor, and usually negative, effect on the obtained Jaccard indicies (Figure S9). Particularly removing the first whole minute weakened the Jaccard indices in almost all cases, and only the brain in the UCB-J data analysed with GMM clearly benefitted from it. However, there were multiple cases when removing either 10 or 30 seconds improved the results for some VOIs and segmentation methods. For example, with GMM the lungs from the F-DPA data had the

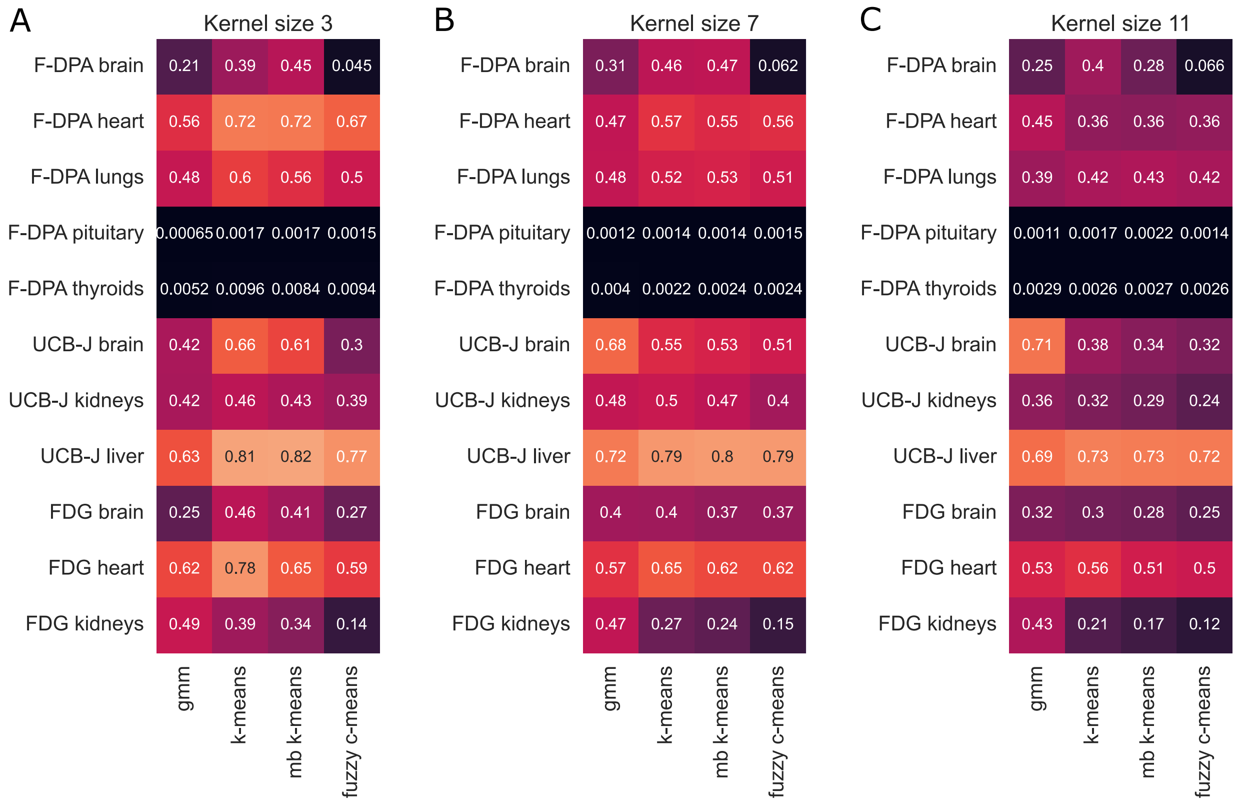

FIGURE S8. Jaccard indices for different VOIs (rows) and segmentation methods (cols) when the data has been preprocessed using median filtering with kernel sizes (A) 3, (B) 7, and (C) 11.

highest mean Jaccard index if the first 10 seconds were removed prior to the clustering. K-means had overall stable performance, but it slightly benefitted from removing 10 and 30 seconds when analysing the brain from the F-DPA and FGD dataset, respectively. For mini-batch k-means the results clearly improved for the heart from the F-DPA dataset and for the kidneys from the FDG dataset if the first 10 seconds were removed. Removing time points did not drastically improve the Jaccard indices of any VOIs if fuzzy c-means was used, but the lungs from the F-DPA data mildly benefitted from it if only the first 10 seconds was removed. As removing early time points did not have systematic positive impact on our results, we continued our analyses using all time points.

Notably, despite the mostly negative or neutral effect of the removal of the first time points on the Jaccard indices, it had also an advantage: the stability of the performance of GMM increased as time points were removed. With the full data GMM threw errors with all FDG images and without the first 10 second, three of the images could be analysed. However, when we removed the first 30 or 60 seconds (i.e. 3 or 6 time points), all but one image worked with GMM. With full data GMM was able to analyse 5 out of 10 F-DPA

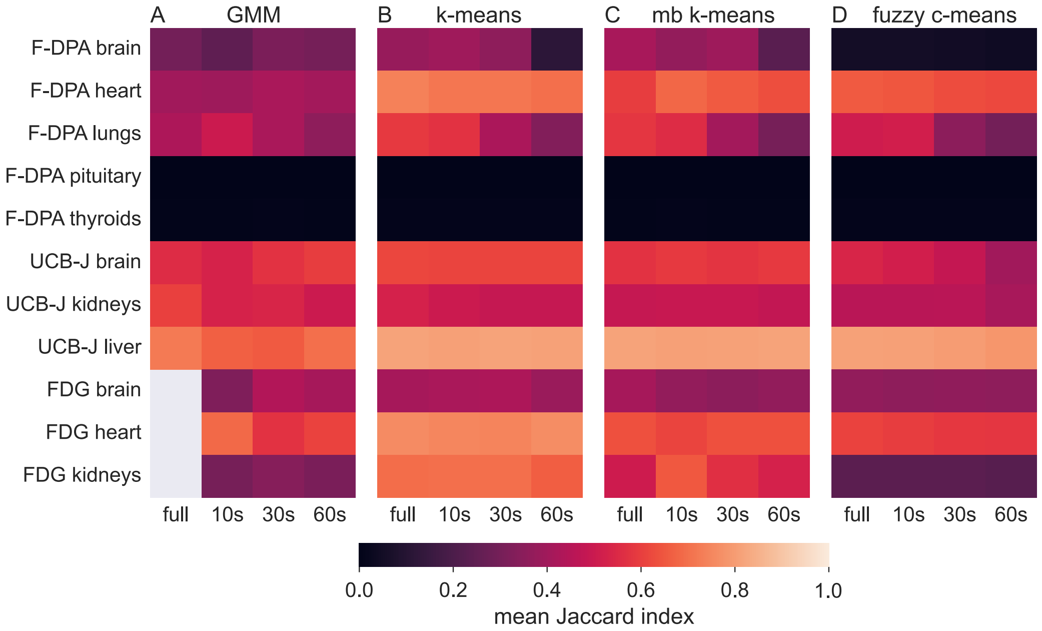

Figure S9. Mean Jaccard indices (rows) of different VOIs after removing the first 0, 10, 30, or 60 seconds (columns) from the data prior to segmentation with (A) GMM, (B) k-means, (C) mini-batch k-means, or (D) fuzzy c-means.

images and 3 out of 10 UCB-J images, but if any time points were removed, it was able to analyse all images from both datasets.

7. Mean Jaccard indices of different preprocessing approaches

Here we present the mean Jaccard indices after different denoising, scaling, and dimensionality reduction techniques from Figure 2 and Figure 3 of the main manuscript in table format, so that the readers interested in the exact numbers can investigate them.

Table S2. Mean Jaccard indices obtained using different preprocessing approaches before the segmentation.

(a) Mean Jaccard indices of different segmentation methods obtained using different denoising approaches. Wave, gaus, med, and nlm refer to wavelet, gaussian, median, and nlmeans denoising, respectively.

	GMM						k-means						mb k-means						fuzzy c-means					
	none	tv	wave.	gaus.	med.	nlm	none	tv	wave.	gaus.	med.	nlm	none	tv	wave.	gaus.	med.	nlm	none	tv	wave.	gaus.	med.	nlm
F-DPA brain	0.11	0.11	0.10	0.29	0.21	0.21	0.09	0.08	0.09	0.38	0.39	0.10	0.08	0.10	0.11	0.41	0.45	0.12	0.03	0.03	0.03	0.05	0.05	0.04
F-DPA heart	0.35	0.23	0.27	0.40	0.56	0.44	0.59	0.59	0.59	0.74	0.72	0.60	0.48	0.51	0.47	0.60	0.72	0.43	0.11	0.11	0.11	0.66	0.67	0.11
F-DPA lungs	0.44	0.39	0.41	0.43	0.48	0.49	0.53	0.53	0.52	0.59	0.60	0.52	0.45	0.48	0.48	0.58	0.56	0.49	0.18	0.19	0.18	0.51	0.50	0.19
F-DPA pituitary	0.00	0.00	0.00	0.00	0.00	0.00	0.00	0.00	0.00	0.00	0.00	0.00	0.00	0.00	0.00	0.00	0.00	0.00	0.00	0.00	0.00	0.00	0.00	0.00
F-DPA thyroid	0.01	0.00	0.01	0.01	0.01	0.01	0.01	0.01	0.01	0.01	0.01	0.01	0.01	0.01	0.01	0.01	0.01	0.01	0.00	0.00	0.00	0.01	0.01	0.00
UCB-J brain	0.30	0.28	0.30	0.56	0.42	0.39	0.50	0.51	0.51	0.61	0.66	0.51	0.42	0.39	0.40	0.57	0.61	0.40	0.15	0.15	0.14	0.54	0.30	0.15
UCB-J kidneys	0.30	0.33	0.28	0.60	0.42	0.34	0.32	0.31	0.32	0.52	0.46	0.32	0.33	0.32	0.33	0.49	0.43	0.33	0.11	0.12	0.12	0.45	0.39	0.12
UCB-J liver	0.61	0.62	0.64	0.72	0.63	0.53	0.78	0.79	0.79	0.81	0.81	0.78	0.77	0.77	0.78	0.81	0.82	0.77	0.69	0.67	0.68	0.81	0.77	0.69
FDG brain	0.13	0.11	0.12	nan	0.25	0.16	0.18	0.18	0.18	0.41	0.46	0.19	0.15	0.17	0.14	0.41	0.41	0.16	0.06	0.05	0.05	0.37	0.27	0.06
FDG heart	0.48	0.39	0.43	nan	0.62	0.56	0.56	0.54	0.54	0.76	0.78	0.57	0.53	0.54	0.49	0.64	0.65	0.54	0.05	0.05	0.05	0.61	0.59	0.05
FDG kidneys	0.35	0.33	0.34	nan	0.49	0.37	0.24	0.25	0.27	0.69	0.39	0.23	0.17	0.18	0.18	0.51	0.34	0.17	0.06	0.06	0.06	0.24	0.14	0.06

(b) Mean Jaccard indices of different segmentation methods obtained using different scaling approaches. Abbreviations z.sc, log, and sum1 refer to z-scoring, logistic scaling, and sum-to-1 scaling as defined in the main manuscript.

	Hierarchical				GMM				k-means				mb k-means				fuzzy c-means				HDBSCAN			
	none	z-sc.	log.	sum1	none	z-sc.	log.	sum1	none	z-sc.	log.	sum1	none	z-sc.	log.	sum1	none	z-sc.	log.	sum1	none	z-sc.	log	sum1
F-DPA brain	0.01	0.01	0.01	0.01	0.29	0.24	0.51	0.26	0.38	0.39	0.59	0.62	0.41	0.38	0.57	0.55	0.05	0.05	0.08	0.04	0.01	0.01	0.29	0.01
F-DPA heart	0.00	0.00	0.00	0.00	0.40	0.42	0.37	0.01	0.74	0.74	0.11	0.03	0.60	0.67	0.11	0.02	0.66	0.64	0.12	0.01	0.05	0.08	0.46	0.00
F-DPA lungs	0.02	0.02	0.02	0.02	0.43	0.50	0.58	0.09	0.59	0.59	0.33	0.44	0.58	0.55	0.32	0.42	0.51	0.51	0.37	0.08	0.02	0.02	0.01	0.02
F-DPA pituitary	0.00	0.00	0.00	0.00	0.00	0.00	0.00	0.00	0.00	0.00	0.00	0.00	0.00	0.00	0.00	0.00	0.00	0.00	0.00	0.00	0.00	0.00	0.00	0.00
F-DPA thyroid	0.00	0.00	0.00	0.00	0.01	0.01	0.01	0.00	0.01	0.01	0.00	0.00	0.01	0.01	0.00	0.00	0.01	0.01	0.00	0.00	0.02	0.03	0.03	0.00
UCB-J brain	0.01	0.01	0.01	0.01	0.56	0.61	0.40	0.04	0.61	0.62	0.25	0.09	0.57	0.57	0.23	0.08	0.54	0.53	0.30	0.02	0.03	0.04	0.13	0.01
UCB-J kidneys	0.01	0.01	0.01	0.01	0.60	0.54	0.54	0.09	0.52	0.55	0.53	0.28	0.49	0.53	0.49	0.25	0.45	0.50	0.42	0.20	0.01	0.02	0.02	0.01
UCB-J liver	0.06	0.06	0.06	0.06	0.72	0.69	0.76	0.18	0.81	0.81	0.67	0.55	0.81	0.80	0.66	0.55	0.81	0.81	0.66	0.42	0.05	0.05	0.16	0.06
FDG brain	0.01	0.01	0.01	0.01	nan	0.24	0.29	0.03	0.41	0.44	0.26	0.08	0.41	0.39	0.20	0.08	0.37	0.41	0.25	0.06	0.01	0.01	0.03	0.01
FDG heart	0.01	0.01	0.01	0.01	nan	0.53	0.56	0.03	0.76	0.78	0.23	0.06	0.64	0.59	0.20	0.07	0.61	0.65	0.23	0.02	0.01	0.01	0.45	0.01
FDG kidneys	0.01	0.01	0.01	0.01	nan	0.32	0.32	0.04	0.69	0.70	0.18	0.08	0.51	0.58	0.17	0.08	0.24	0.21	0.19	0.07	0.02	0.01	0.03	0.01

(c) Mean Jaccard indices of different segmentation methods obtained using different dimensionality reduction approaches.

	Hierarchical				GMM					k-means					mb k-means					fuzzy c-means				HDBSCAN			
	none	t-SVD	PCA	ICA	none	t-SVD	PCA	ICA	pPCA	none	t-SVD	PCA	ICA	pPCA	none	t-SVD	PCA	ICA	pPCA	none	t-SVD	PCA	ICA	none	t-SVD	PCA	ICA
F-DPA brain	0.01	0.01	0.01	0.01	0.24	0.31	0.32	0.08	0.10	0.39	0.36	0.35	0.19	0.08	0.38	0.42	0.45	0.19	0.09	0.05	0.05	0.05	0.04	0.01	0.01	0.01	0.01
F-DPA heart	0.00	0.00	0.00	0.00	0.42	0.46	0.46	0.44	0.20	0.74	0.71	0.71	0.29	0.63	0.67	0.62	0.60	0.23	0.49	0.64	0.63	0.64	0.02	0.08	0.02	0.02	0.01
F-DPA lungs	0.02	0.02	0.02	0.02	0.50	0.50	0.50	0.39	0.27	0.59	0.58	0.58	0.45	0.54	0.55	0.54	0.57	0.44	0.51	0.51	0.53	0.52	0.08	0.02	0.02	0.02	0.02
F-DPA pituitary	0.00	0.00	0.00	0.00	0.00	0.00	0.00	0.00	0.00	0.00	0.00	0.00	0.00	0.00	0.00	0.00	0.00	0.00	0.00	0.00	0.00	0.00	0.00	0.00	0.00	0.00	0.00
F-DPA thyroid	0.00	0.00	0.00	0.00	0.01	0.01	0.01	0.00	0.01	0.01	0.01	0.01	0.00	0.01	0.01	0.01	0.01	0.00	0.01	0.01	0.01	0.01	0.00	0.03	0.00	0.00	0.00
UCB-J brain	0.01	0.01	0.01	0.01	0.61	0.56	0.57	0.45	0.26	0.62	0.62	0.62	0.50	0.45	0.57	0.58	0.54	0.45	0.28	0.53	0.54	0.53	0.04	0.04	0.02	0.01	0.01
UCB-J kidneys	0.01	0.01	0.01	0.01	0.54	0.54	0.51	0.52	0.22	0.55	0.55	0.55	0.40	0.43	0.53	0.52	0.52	0.37	0.44	0.50	0.50	0.50	0.04	0.02	0.01	0.01	0.01
UCB-J liver	0.06	0.06	0.06	0.06	0.69	0.69	0.70	0.68	0.52	0.81	0.81	0.80	0.59	0.77	0.80	0.79	0.80	0.55	0.76	0.81	0.81	0.81	0.17	0.05	0.05	0.06	0.03
FDG brain	0.01	0.01	0.01	0.01	0.24	0.29	0.35	0.03	0.09	0.44	0.43	0.43	0.10	0.27	0.39	0.43	0.37	0.10	0.27	0.41	0.41	0.40	0.04	0.01	0.01	0.01	0.01
FDG heart	0.01	0.01	0.01	0.01	0.53	0.56	0.60	0.62	0.30	0.78	0.77	0.78	0.48	0.61	0.59	0.61	0.64	0.45	0.56	0.65	0.62	0.63	0.02	0.01	0.01	0.01	0.01
FDG kidneys	0.01	0.01	0.01	0.01	0.32	0.34	0.33	0.46	0.31	0.70	0.70	0.72	0.23	0.56	0.58	0.53	0.52	0.17	0.42	0.21	0.23	0.22	0.02	0.01	0.01	0.01	0.00

8. Combinations of different preprocessing types

In the main manuscript we first tested if denoising the images improves the results and then proceeded to test different scaling approaches on the denoised data. Similarly different dimensionality reduction methods were evaluated using images that were denoised and scaled. This does not reveal if, for example, scaling alone would provide weaker or stronger results than denoising alone, or if dimensionality reduction benefits from denoising or not. Here we test the effect of different preprocessing combinations, where gaussian filtering represents denoising, z-score scaling, and PCA dimensionality reduction.

Our results show that denoising is the most important preprocessing step as it clearly improves the mean Jaccard indices if done alone or combined with scaling and/or dimensionality reduction (Figure S10, Table S3). In fact, the average improvement of denoising over the segmented organs was over 50 % with all the tested methods. In smaller scale, k-means and mini batck k-means results also benefit from scaling weather combined with other preprocessing steps or not. Unsurprisingly, dimensionality reduction (represented by PCA) slightly benefitted from scaling (Figure S10). The benefits of dimensionality reduction alone are related to running time, not to the performance of the method (Table S3).

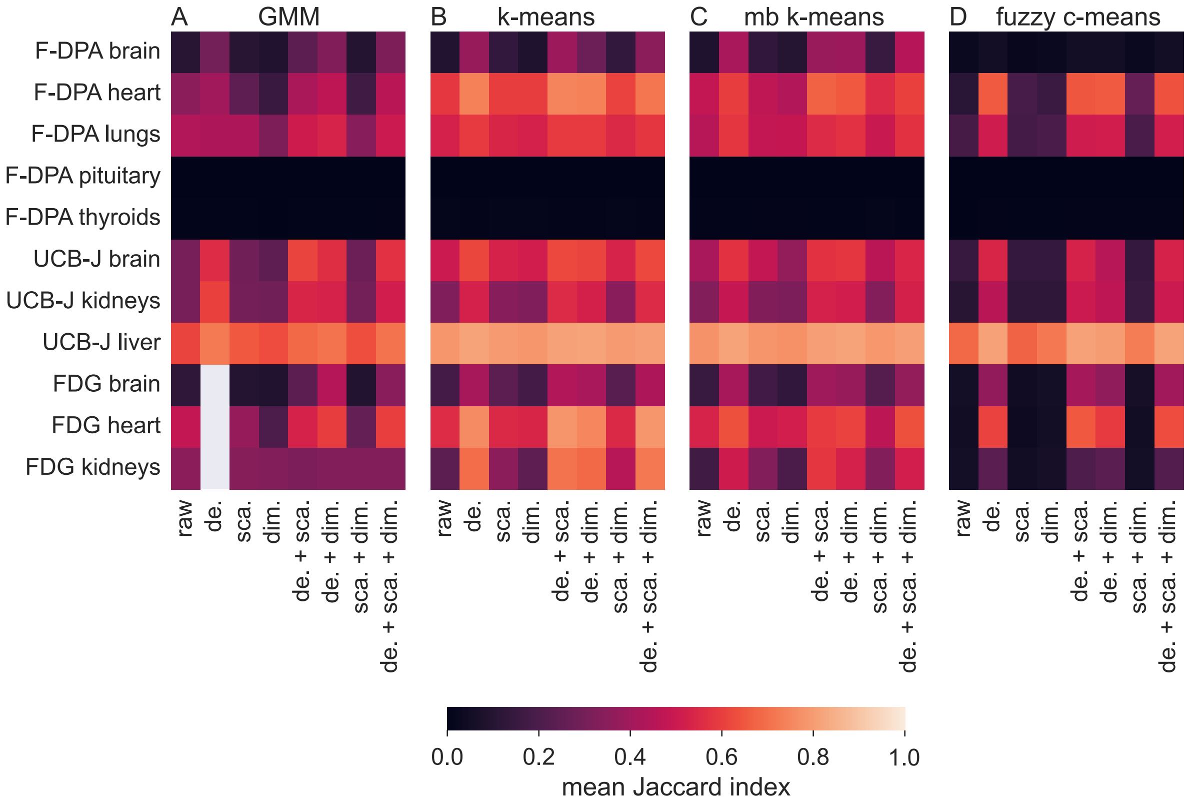

Figure S10. Mean Jaccard indices for different VOIs (rows) using different preprocessing combinations (columns) from (A) Gaussian mixture model, (B) k-means, (C) mini-batch k-means, and (D) fuzzy c-means. Column labels de., sca., and dim. refer to denoising, scaling, and dimensionality reduction, respectivelty.

9. Number of principal components

Selecting a suitable number of principal components to use for dimensionality reduction can affect the outcome as using too few components may not capture moderate differences in signals and using too many unnecessarily increases the running time. We tested how using different number components affects the Jaccard indices of different VOIs for different segmentation methods. Initially we tested 5, 10, ..., 45 components, but as the smaller end of our scale provided the best performance, we added component numbers 2, 3, 4, 6, and 8 to this evaluation.

For most methods the number of used principal components did not have high impact on the average Jaccard indices as long as the used number of components was above three (Figure S11). An exception to that was GMM, which systematically benefitted from 3-6 principal components, after which the performance systematically weakened when the number of PCAs increased. K-means had very stable performance without

Table S3. The effect of individual preprocessing step on the mean Jaccard indices measured by change percent. The change is calculated by dividing the difference in mean Jaccard indices obtained with one preprocessing step and without any preprocessing by the mean Jaccard obtained without any preprocessing (column 'raw').

	GMM				k-means			
Organ	raw	denoise%	scale%	dim.red.%	raw	denoise%	scale%	dim.red.%
F-DPA brain	0.11	1.75	-0.01	-0.18	0.09	3.14	0.48	-0.09
F-DPA heart	0.35	0.14	-0.31	-0.56	0.59	0.26	0.02	0.02
F-DPA lungs	0.44	-0.02	-0.03	-0.27	0.53	0.12	0.02	0.00
F-DPA pituitary	0.00	0.08	0.24	-0.21	0.00	0.02	0.19	0.05
F-DPA thyroid	0.01	0.17	0.05	-0.32	0.01	-0.32	-0.07	0.00
UCB-J brain	0.30	0.84	-0.04	-0.18	0.50	0.22	0.05	0.02
UCB-J kidneys	0.30	1.00	0.00	-0.05	0.32	0.63	0.06	0.00
UCB-J liver	0.61	0.18	0.07	0.02	0.78	0.04	0.01	0.00
FDG brain	0.13	nan	-0.25	-0.30	0.18	1.26	0.32	0.00
FDG heart	0.48	nan	-0.22	-0.58	0.56	0.37	-0.02	-0.03
FDG kidneys	0.35	nan	-0.05	-0.07	0.24	1.91	0.46	0.03
mean change		0.52	-0.05	-0.25		0.70	0.14	0.00

	mb k-means				fuzzy c-means			
Organ	raw	denoise%	scale%	dim.red.%	raw	denoise%	scale%	dim.red.%
F-DPA brain	0.08	3.94	0.56	0.19	0.03	0.85	-0.19	0.04
F-DPA heart	0.48	0.24	-0.02	-0.09	0.11	4.85	0.69	0.42
F-DPA lungs	0.45	0.28	0.06	0.07	0.18	1.76	-0.04	0.05
F-DPA pituitary	0.00	0.09	-0.02	-0.09	0.00	0.64	-0.20	0.00
F-DPA thyroid	0.01	-0.29	-0.02	-0.09	0.00	1.29	0.50	0.29
UCB-J brain	0.42	0.37	0.15	-0.11	0.15	2.65	-0.02	-0.02
UCB-J kidneys	0.33	0.48	0.00	-0.04	0.11	3.17	0.14	0.15
UCB-J liver	0.77	0.05	0.01	0.00	0.69	0.18	-0.02	0.04
FDG brain	0.15	1.73	0.14	-0.13	0.06	5.45	-0.20	-0.02
FDG heart	0.53	0.19	-0.06	-0.03	0.05	11.02	-0.29	0.04
FDG kidneys	0.17	1.95	0.92	0.15	0.06	3.04	-0.19	0.04
mean change		0.82	0.16	-0.02		3.17	0.02	0.09

considerable trend for the number of principal components, whereas mini batck k-means had unstable performance, but without systematic trend, if at least 4 PCAs was used. While most VOIs were unaffected by the number of principal components (> 3) in the fuzzy c-means analysis, some VOIs, such as the brain and the kidneys in different datasets, mildly benefitted from small number of components. GMM, k-means, and fuzzy c-means

reached their best average performance with 4-6 principal components, so we claim 5 as the optimal number of components for this study.

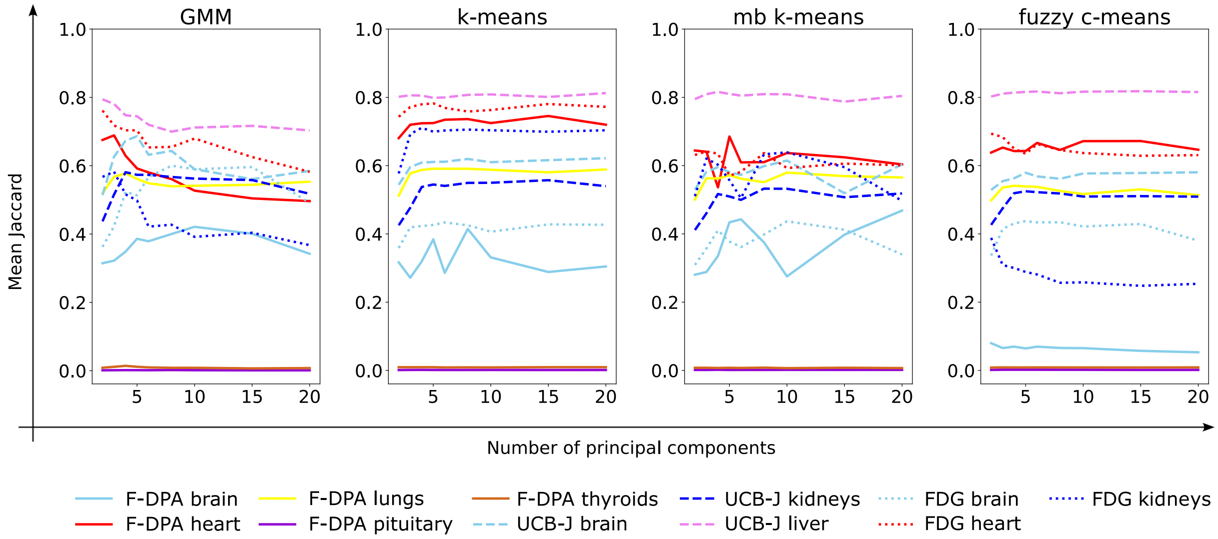

Figure S11. Mean Jaccard indices for different VOIs and segmentation methods as a function of the number of used principal components. Component numbers above 20 were excluded from the figure as they did not provide new information, but made it difficult to interpret the results for small numbers of components.

10. Validation data examples

Here we present and visualise more examples of the segmentation results on the validation data than fitted into the main manuscript. Besides the very small VOIs of the thyroid glands and the pituitary gland, also the brain was difficult to segments from the F-DPA data, likely due to its often low signal. Indeed, in many cases the best matching cluster was a huge scattered cluster. Fuzzy c-means was the most prone method to this issue and GMM the least (Figure S12). The lungs typically clustered nicely together despite the boarders being often a bit undefined, whereas the heart was usually otherwise connected segment, but often included voxels from the kidneys (Figure S12). In the UCB-J data voxels from the liver typically clusters well together with median Jaccard index ranging from 0.749 to 0.813 for different methods (section 3.4 of the main manuscript). However, in some cases small parts of the liver cluster together with the brain (Figure S13). Excluding this issue, the brain clusters well in most UCB-J images. The kidneys were the hardest VOI to segment from the UCB-J dataset and particularly fuzzy c-means often clustered voxels from the heart with the kidneys (Figure S13). From the FDG dataset, the heart typically clustered otherwise well, but often the cluster included voxels also from the Harderian glands and kidneys. K-means and especially GMM managed to exlude these extra voxels better than mini-batch k-means or fuzzy c-means (Figure S14). While k-means had the highest median Jaccard for the heart, GMM segmented one connected, but often a bit too large, area without many spatially detached voxels. Notably, the brain from the FDG images has previously been reported to cluster together with the brown adipo tissue in rats' neck [11], but with this preprocessed data, this did not happen. Instead, the brain often clusters together with parts of the salivary glands (Figure S14).

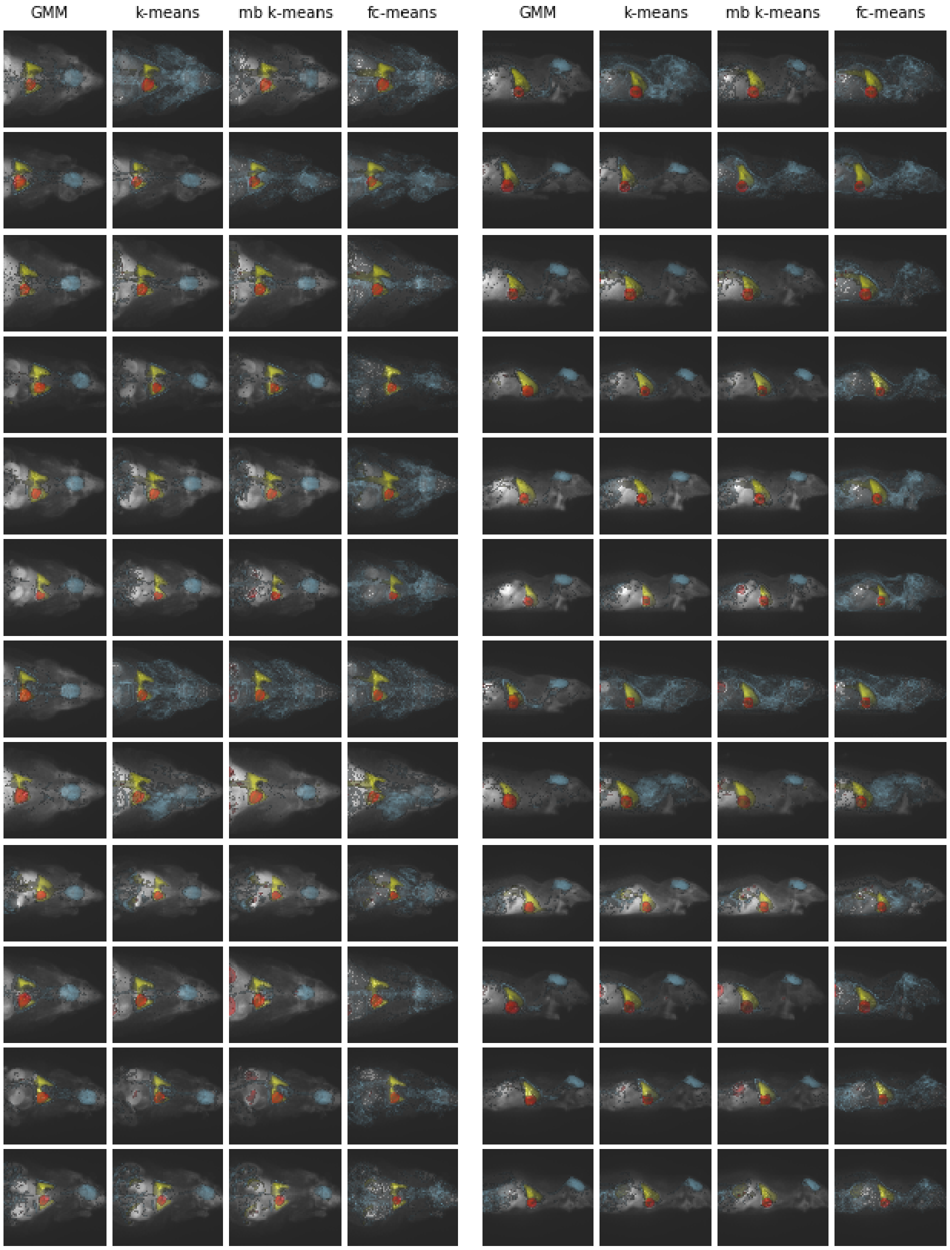

Figure S12. Clusters corresponding to the analysed VOIs from 12 randomly selected images from the F-DPA dataset (rows) obtained using different methods (columns). The clusters for the brain (cyan), the heart (red), and the lungs (yellow) are drawn on top of the corresponding original PET images. The thyroid glands and the pituitary gland are missing because they were typically within a very large and sparse cluster, and viasualising those made the figure hard to read.

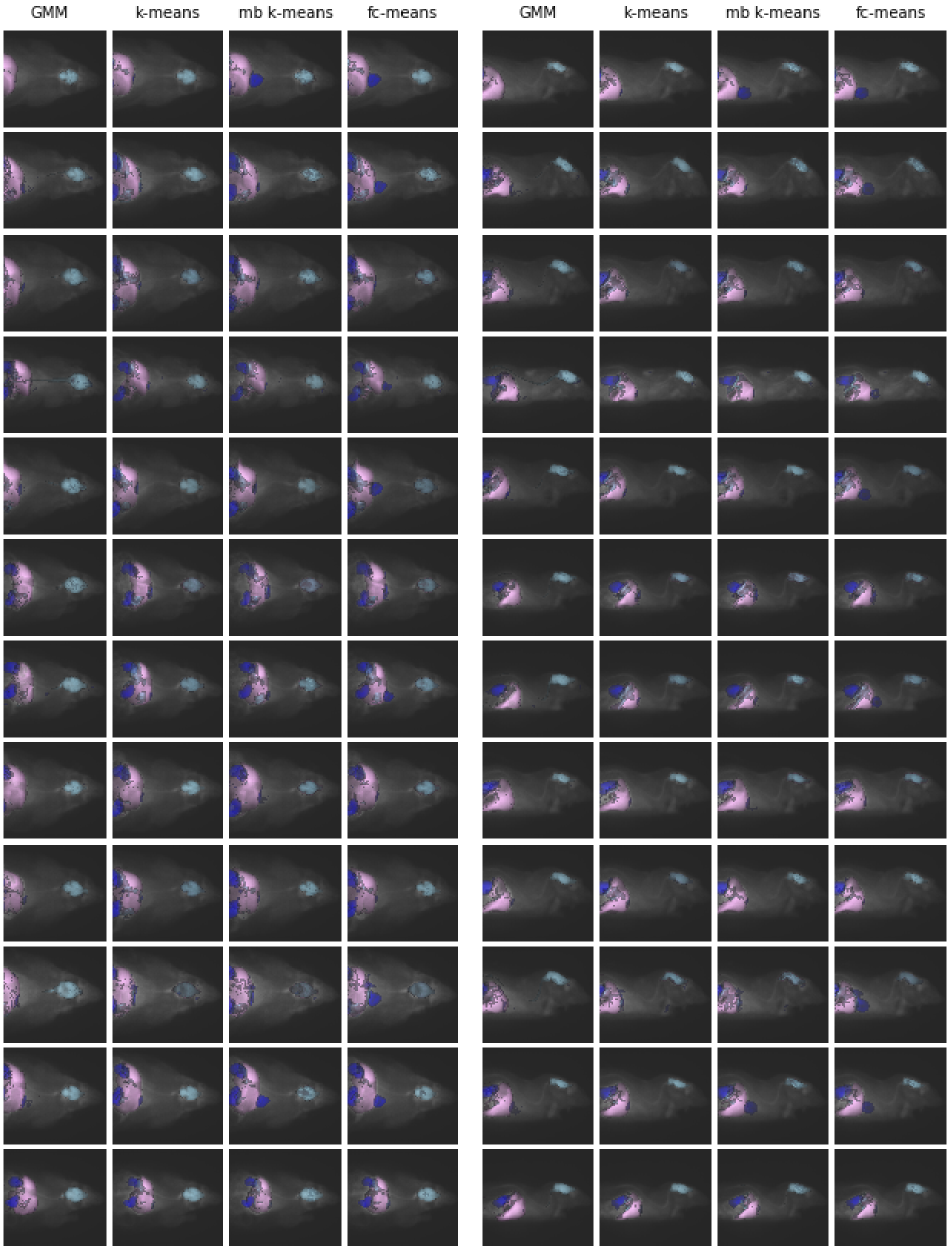

Figure S13. Clusters corresponding to the analysed VOIs from 12 randomly selected images from the UCB-J dataset (rows) obtained using different methods (columns). The clusters for the brain (cyan), the liver (violet), and the kidneys (blue) are drawn on top of the corresponding original PET images.

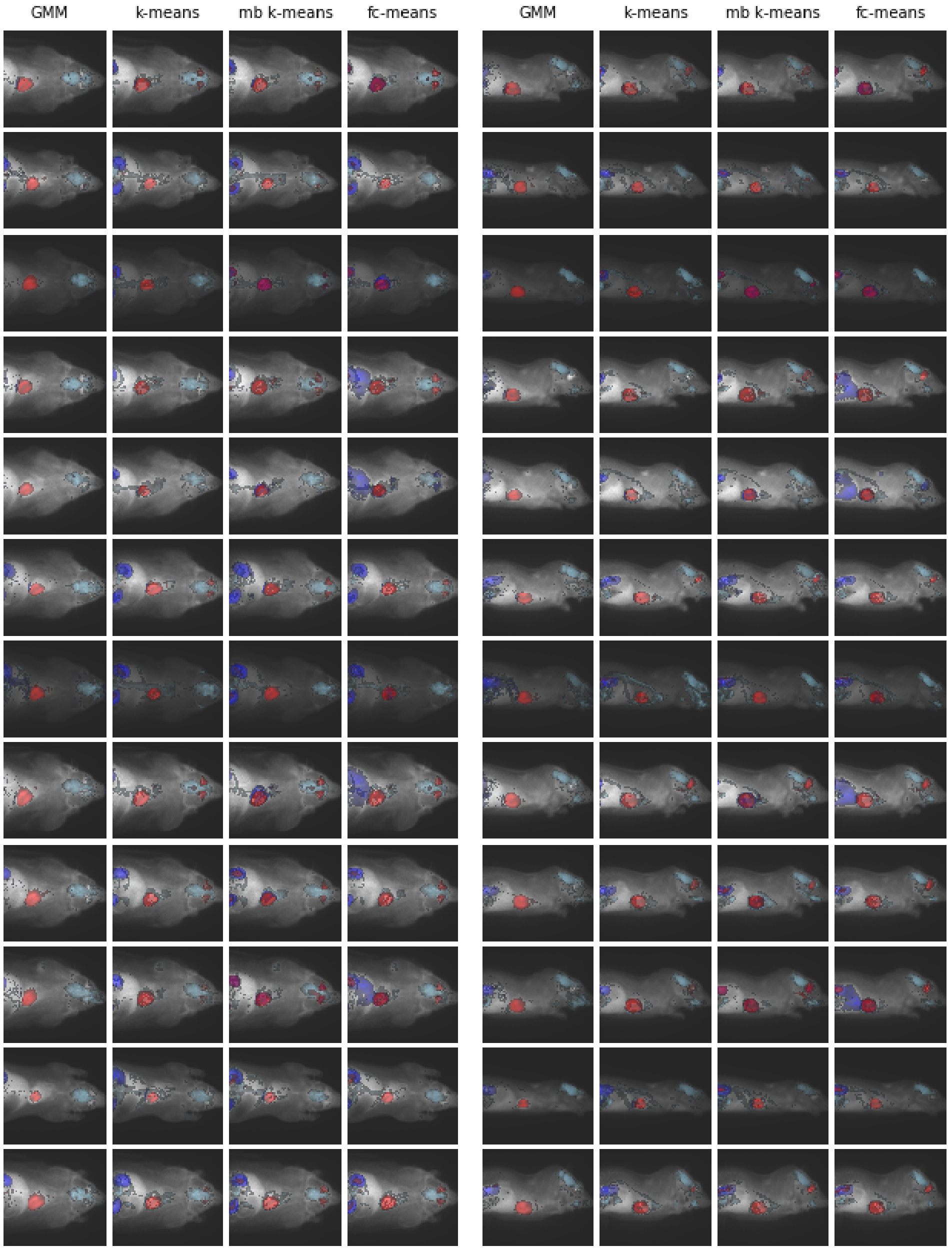

Figure S14. Clusters corresponding to the analysed VOIs from 12 randomly selected images from the FDG dataset (rows) obtained using different methods (columns). The clusters for the brain (cyan), the heart (red), and the lungs (yellow) are drawn on top of the corresponding original PET images.

11. Small images

We wanted to study if the excluded resource intensive methods would perform well, if they were usable from practical point of view, so we run them on very small dynamic 4D images. With the small images, also methods BIRCH, mean shift, spectral clustering, DBSCAN, slic, watershed, and random walker became usable. This experiment also serves other purpose: the small sub-images to be segmented are selected so that the pituitary gland is included in them. Thus we can test if the small VOIs that are difficult to segment with the tested unsupervised methods can be detected if the input image is split into smaller parts.

The segmentation of the pituitary gland improved by splitting the input image, but still remained difficult as the median Jaccard index was below 0.05 for all methods except slic, which had the median Jaccard of 0.125. Figure S15 visualises the segmentation results for the image with the highest Jaccard index for the pituitary gland. In that image, the pituitary gland had higher intensity than in most cases (Figure S16). Among the methods not usable with the full size images, BIRCH performed similarly to GMM, k-means, mini-batch k-means, and fuzzy c-means by providing somewhat equally sized segments. Spectral clustering provided the most intuitive segmentation among the clustering methods with uneven output clusters. Besides the clustering methods, three other segmentation approaches were usable with the small images, slic being the most promising among them, while Watershed and random walker provided unintuitive segmentation at least without further parameter optimisation (Figures S15 and S16).

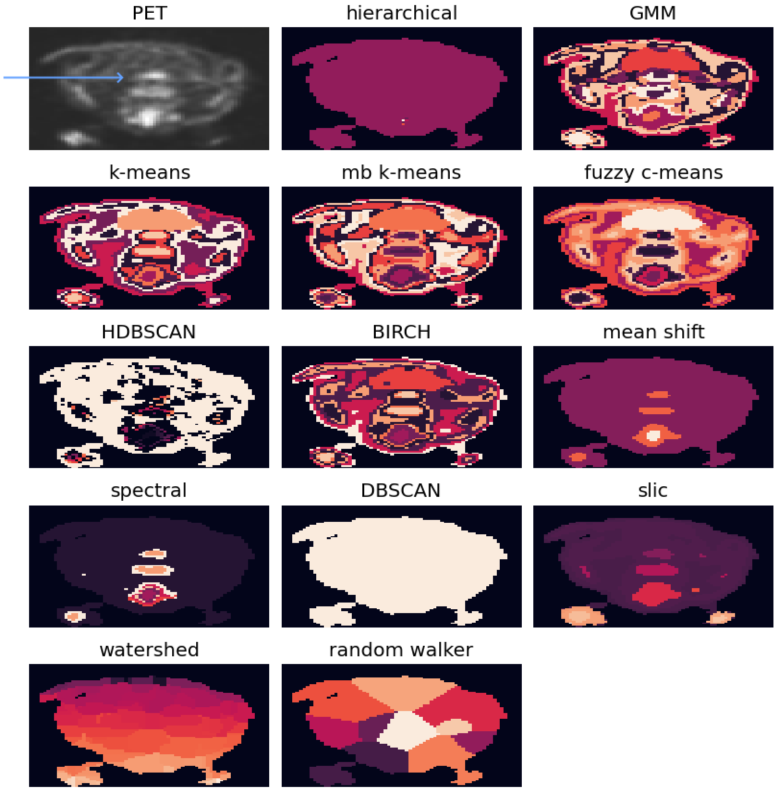

Figure S15. Segmentations of the middle slice from the F-DPA image with the highest median Jaccard index. Top left corner visualises the corresponding slice of the PET image prior to preprocessing (sum over time points) and the blue arrow indicates the pituitary gland in it.

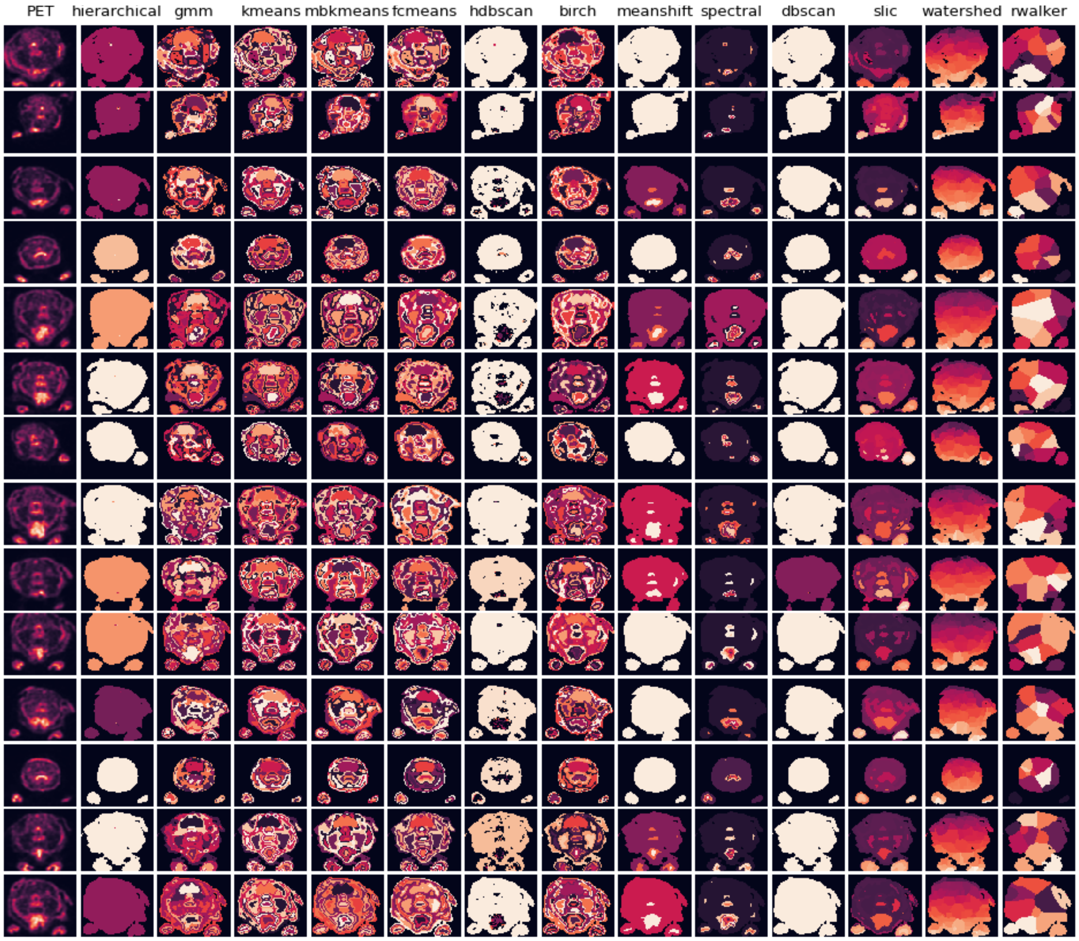

FIGURE S16. Segmentation of the middle slice by different methods (cols) for 14 randomly selected example images from F-DPA data (rows). The first column is the original PET image (sum over time points) prior preprocessing. 30 voxels are cropped from each border for this visualisation.

12. Human images

Here we present a visualisation of the segmentation of all analysed human images (Figure S17) as only one human subject per dataset fitted into the main manuscript. In all cases for example brain areas cluster separately, and the segmentation gives a nice overview of the images while the very high contast in some Radiowater images (the radioactivity within the injection vein shadows everything else) makes them hard to interpret without any other visual aid.

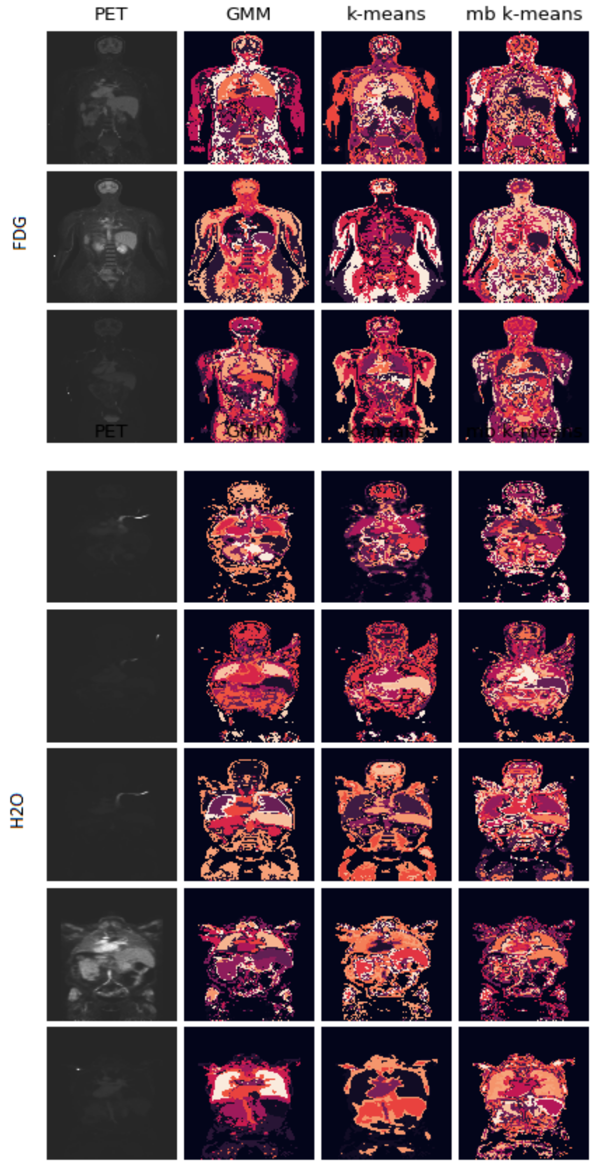

Figure S17. The segments of the middle slice of all analysed human images (rows). The left-most column visualised the corresponding slice of the original PET image prior to preprocessing (sum over time points) and the remaining columns illustrate the segmentations by different methods.
